# Role of human Kallistatin in glucose and energy homeostasis in mice

**DOI:** 10.1016/j.molmet.2024.101905

**Published:** 2024-02-29

**Authors:** Leontine Sandforth, Sebastian Brachs, Julia Reinke, Diana Willmes, Gencer Sancar, Judith Seigner, David Juarez-Lopez, Arvid Sandforth, Jeffrey D. McBride, Jian-Xing Ma, Sven Haufe, Jens Jordan, Andreas L. Birkenfeld

**Affiliations:** 1 Internal Medicine IV, Endocrinology, Diabetology and Nephrology, University Hospital of Tuebingen, Tuebingen, Germany; 2 Institute of Diabetes Research and Metabolic Diseases (IDM) of the Helmholtz Center Munich, Tuebingen, Germany; 3 German Center for Diabetes Research (DZD), Neuherberg, Germany; 4 Department of Endocrinology and Metabolism, Charité – Universitätsmedizin Berlin, corporate member of Freie Universität Berlin and Humboldt-Universität zu Berlin, Berlin, Germany; 5 DZHK (German Centre for Cardiovascular Research), partner site Berlin, Germany; 6 Section of Metabolic Vascular Medicine, Department of Medicine III, University Clinic Dresden, TU Dresden, Germany; 7 Department of Physiology, University of Oklahoma Health Sciences Center, Oklahoma City, OK, USA; 8 Department of Biochemistry, Wake Forest University School of Medicine, Winston–Salem, NC 27157, USA; 9 Department of Rehabilitation and Sports Medicine, Hannover Medical School (MHH), Hannover, Germany; 10 Institute of Aerospace Medicine, German Aerospace Center (DLR), Cologne, Germany; 11 Medical Faculty, University of Cologne, Cologne, Germany; 12 Department of Diabetes, Life Sciences & Medicine, Cardiovascular Medicine & Life Sciences, King's College London, UK

**Keywords:** Type 2 diabetes, Diet-induced insulin resistance, Kallistatin, SERPIN A4

## Abstract

**Objective:**

Kallistatin (KST), also known as SERPIN A4, is a circulating, broadly acting human plasma protein with pleiotropic properties. Clinical studies in humans revealed reduced KST levels in obesity. The exact role of KST in glucose and energy homeostasis in the setting of insulin resistance and type 2 diabetes is currently unknown.

**Methods:**

Kallistatin mRNA expression in human subcutaneous white adipose tissue (sWAT) of 47 people with overweight to obesity of the clinical trial “Comparison of Low Fat and Low Carbohydrate Diets With Respect to Weight Loss and Metabolic Effects (B-SMART)” was measured. Moreover, we studied transgenic mice systemically overexpressing human KST (hKST-TG) and wild type littermate control mice (WT) under normal chow (NCD) and high-fat diet (HFD) conditions.

**Results:**

In sWAT of people with overweight to obesity, *KST* mRNA increased after diet-induced weight loss. On NCD, we did not observe differences between hKST-TG and WT mice. Under HFD conditions, body weight, body fat and liver fat content did not differ between genotypes. Yet, during intraperitoneal glucose tolerance tests (ipGTT) insulin excursions and HOMA-IR were lower in hKST-TG (4.42 ± 0.87 AU, WT vs. 2.20 ± 0.27 AU, hKST-TG, p < 0.05). Hyperinsulinemic euglycemic clamp studies with tracer-labeled glucose infusion confirmed improved insulin sensitivity by higher glucose infusion rates in hKST-TG mice (31.5 ± 1.78 mg/kg/min, hKST-TG vs. 18.1 ± 1.67 mg/kg/min, WT, p < 0.05). Improved insulin sensitivity was driven by reduced hepatic insulin resistance (clamp hepatic glucose output: 7.7 ± 1.9 mg/kg/min, hKST-TG vs 12.2 ± 0.8 mg/kg/min, WT, p < 0.05), providing evidence for direct insulin sensitizing effects of KST for the first time. Insulin sensitivity was differentially affected in skeletal muscle and adipose tissue. Mechanistically, we observed reduced Wnt signaling in the liver but not in skeletal muscle, which may explain the effect.

**Conclusions:**

*KST* expression increases after weight loss in sWAT from people with obesity. Furthermore, human KST ameliorates diet-induced hepatic insulin resistance in mice, while differentially affecting skeletal muscle and adipose tissue insulin sensitivity. Thus, KST may be an interesting, yet challenging, therapeutic target for patients with obesity and insulin resistance.

## Highlights

• Kallistatin is expressed in human subcutaneous white adipose tissue.
• Kallistatin mRNA expression increases by weight loss in humans with obesity.
• Human Kallistatin improves hepatic insulin sensitivity in diet induced obese mice.
• Kallistatin may contribute to the beneficial metabolic effects of weight loss.

## 1 Introduction

Serine proteinase inhibitors (serpins) affect various physiological processes such as coagulation, fibrinolysis, the complement cascade and arterial blood pressure by binding to their serine proteinases [1,2]. Inhibition of these proteinases is mediated via an irreversible destruction mechanism by rapid insertion of the reaction center loop (RCL) into β-sheet A after cleavage at the scissile P1–P1’ bond, thereby translocating the covalently bound protease to the bottom of the molecule where it is prevented from further functioning by distortion of the active site [3,4]. In this manner, Kallistatin (KST) encoded by *SERPINA4* inhibits multiple proteases, among them kallikrein-related peptidase 7 (KLK7) which is important for epithelial cell shedding and counteracts adipose tissue inflammation [[5], [6], [7]]. The inhibitory effects of KST on tissue kallikreins via its active site can be blocked by binding to heparin via its heparin-binding site [8].

Moreover, KST has pleiotropic functions independent of serine proteinase inhibition. For example, KST blocks signaling cascades including the vascular endothelial growth factor (VEGF), tumor necrosis factor α (TNF-α), High mobility group box protein 1 (HMGB1), and transforming growth factor-β (TGF-β) pathways [9], exerting anti-inflammatory actions. Importantly, KST has also been shown to be a canonical inhibitor of Wnt/β-catenin signaling via the Wnt co-receptor low-density lipoprotein receptor-related protein (LRP6) [2]. Together, these mechanisms have the potential to be beneficial in metabolic diseases such as obesity and type 2 diabetes (T2D). In line with this notion, serum KST levels in healthy African-American adolescents were inversely associated with obesity and atherogenic lipid profiles [10]. In patients with T2D, however, elevated KST levels were associated with impaired wound healing due to diabetic microvascular complications [11], suggesting that KST may have differential effects depending on the disease state.

In mice, adenoviral injection of human KST (hKST) into epididymal adipose tissue reduced body weight gain and improved glucose tolerance, while affecting systemic insulin sensitivity in one study [12] but not the other [13]. In contrast, when hKST was injected into inguinal adipose tissue, body weight as well as glucose metabolism were not affected, suggesting that the observed phenotype was secondary to reduced body weight gain or body fat distribution in the first model [13]. The mechanisms driving reduced body weight gain or body fat distribution remained elusive. Thus, more detailed insight into the role of hKST in metabolic regulation is needed given the potential therapeutic implication.

To shed further light into the role of hKST in systemic regulation of energy, glucose and lipid metabolism, we determined how a lifestyle intervention leading to weight loss altered *KST* expression in subcutaneous adipose tissue in humans with overweight and obesity. We report that the weight loss intervention led to increased *KST* expression in humans. We then went on to analyze how systemic overexpression of hKST in a lean and a diet induced obese (DOI) mouse model affects energy and glucose metabolism. Our models allowed us to investigate systemic, organ-specific, and cellular mechanisms driving systemic metabolic effects of hKST. We show that hKST improved hepatic and systemic insulin sensitivity by means of tracer-based hyperinsulinemic euglycemic (HE) clamps and that this effect was independent of changes in body weight. Moreover, we rule out that hKST drove the effect by inhibition of a low-grade meta-inflammatory process. Finally, we demonstrate that systemic elevation of hKST differentially affected hepatic, skeletal muscle, and adipose tissue glucose handling. We propose that the beneficial hepatic effect of hKST was mediated, at least in part, via inhibition of the Wnt/β-catenin pathway. Together with our observations in humans, our data may serve as a guide for decision making in a potential use of hKST as a therapeutic option in diet-induced obesity and insulin resistance.

## 2 Results

### 2.1 Adipose tissue hKST expression increases after weight loss in overweight to obese individuals

**Figure 1:**
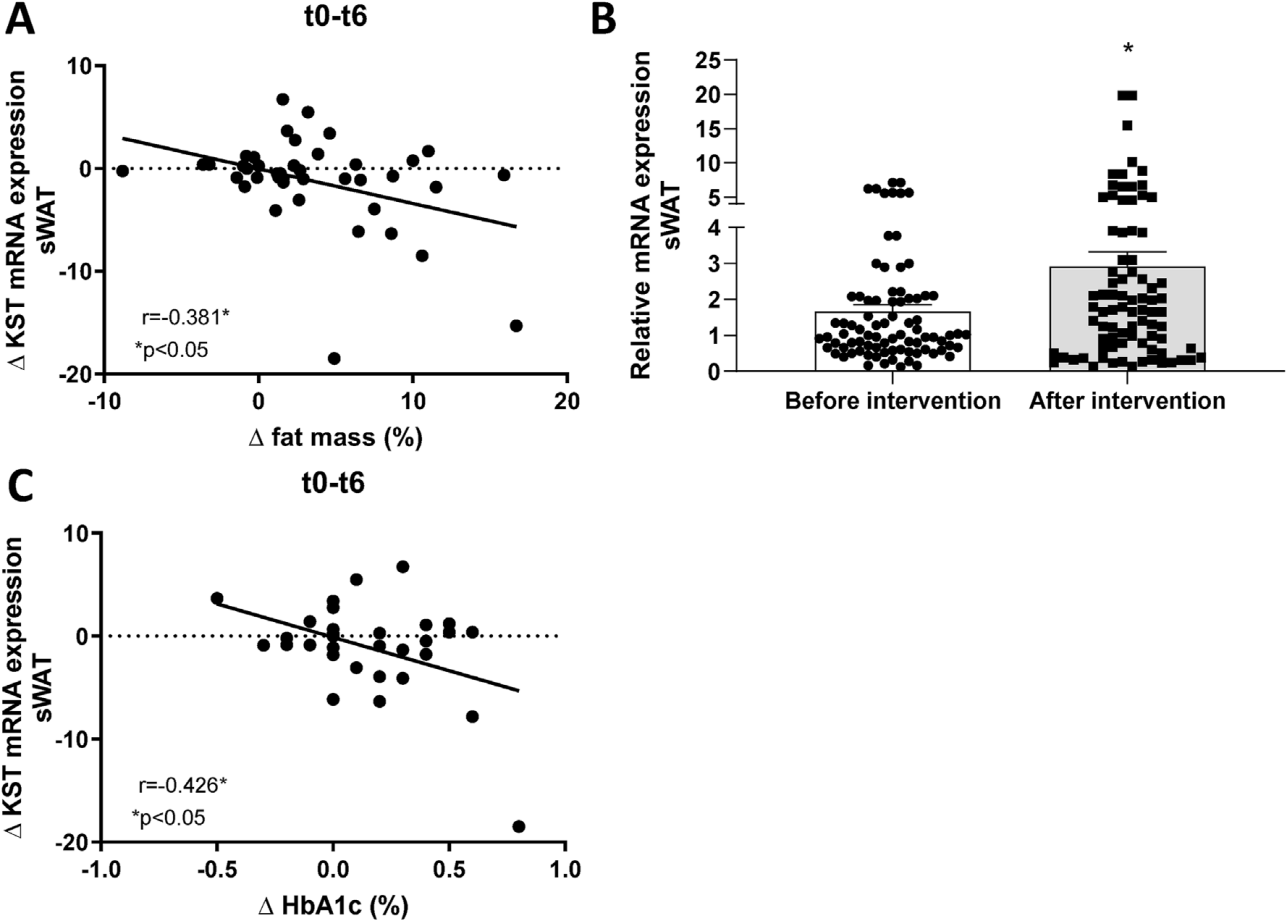
**Increased KST expression after weight loss in people with overweight and obesity.** (A) Correlation of delta (Δ, t0-t6) KST expression with Δ (t0-t6) fat mass (n = 42). (B) KST expression levels before and after intervention (n = 47). (C) Correlation of Δ (t0-t6) KST expression with Δ (t0-t6) HbA1c (n = 33). Data represents mean ± SEM. ∗P < 0.05.

We determined *KST* mRNA expression in abdominal subcutaneous white adipose tissue (sWAT) of 47 patients with overweight to obesity with normal glucose tolerance before and after 6 months of dietary intervention from a previously published human cohort (B-SMART study). Clinical characteristics of participants are listed in Supplementary Table 1. The complete table can be found in the original publication [14]. Our data show that sWAT *KST* mRNA expression is negatively associated with the change in fat mass during the dietary intervention (Figure 1A) and a mean weight loss of 7.7 ± 0.7% and fat loss of 6.6 ± 1.6% due to dietary intervention leads to a more than 1.5-fold increase of sWAT *KST* mRNA expression (Figure 1B). These data show that *KST* expression in adipose tissue increases in subjects with excess adipose tissue with clinically meaningful weight loss [14,15]. Additionally, the change in sWAT *KST* mRNA expression showed a negative correlation with the change in HbA1c (Figure 1C).

### 2.2 Effect of hKST in normal weight, insulin sensitive mice

Given the increase in hKST expression in adipose tissue of patients after weight loss, we next sought to understand whether and how hKST is involved in metabolic control. We used a transgenic mouse model systemically overexpressing hKST (hKST-TG), which previously showed that hKST exerts important effects in mice, including blood pressure regulation [16]. In our study, all comparisons were done between male hKST-TG mice and male wild type littermate controls (WT) under the same dietary intervention.

**Figure 2:**
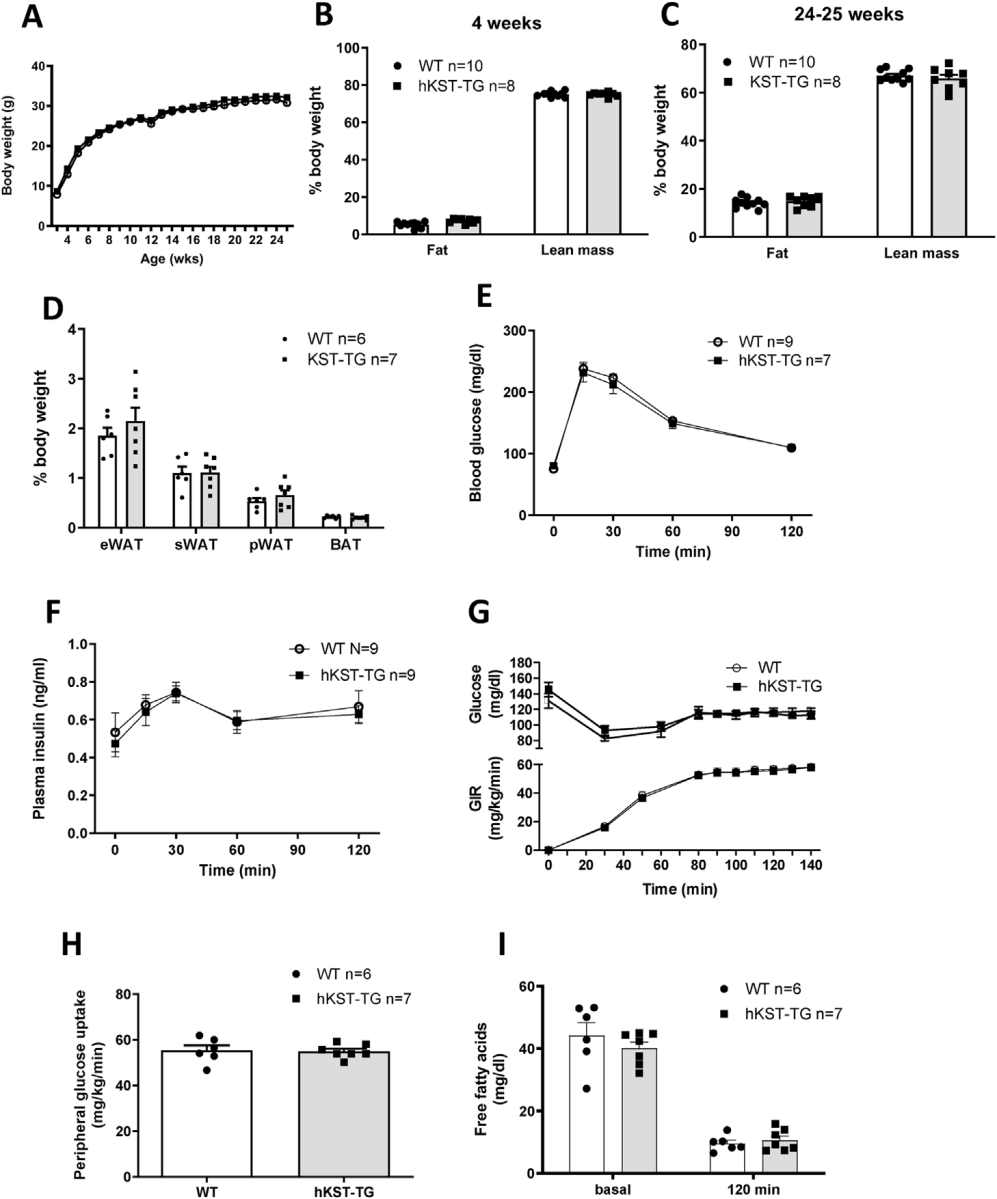
**Body weight and body composition in NCD fed mice.** (A) Development of body weight over 24 weeks. (B) Body fat and (C) lean mass measured by ^1^H nuclear magnetic resonance (NMR) at 4 and 24–25 weeks of age. (D) Weight of epigonadal white adipose tissue (eWAT), subcutaneous white (sWAT), perirenal white (pWAT) and brown adipose tissue (BAT). (E) Blood glucose level and (F) plasma insulin level during ipGTT in 12-week-old animals. (G) Glucose infusion rate (GIR), (H) peripheral glucose uptake and, (I) free fatty acids (FFA) during hyperinsulinemic-euglycemic clamp in 26-week-old mice. Data represents mean ± SEM (n = 6–10).

Body weight of hKST-TG mice and WT littermate controls was measured weekly from 4 to 24–25 weeks of age. hKST-TG mice on NCD did not show differences in body weight compared to WT (Figure 2A). Body fat and lean mass were determined using ^1^H-NMR at 4 and 24–25 weeks (Figure 2B,C). At 4 weeks, we observed a moderately higher body fat content in hKST-TG which did not reach statistical significance and disappeared until 25 weeks, and no differences in lean mass (4 weeks: fat content 5.24 ± 0.49% in WT vs. 7.26 ± 0.43% in hKST-TG, P < 0.05, lean mass: 75.05 ± 0.45% in WT vs. 75.00 ± 0.43% in hKST, P = 0.97; 24 weeks: fat content 14.21 ± 0.66% in WT vs. 14.82 ± 0.78% in hKST-TG, P = 0.47, lean mass: 67.08 ± 0.77% in WT vs. 65.89 ± 1.56% in hKST, P = 0.69). Also, the epigonadal, perirenal and sWAT as well as brown adipose tissue weight did not differ between genotypes (Figure 2D). Overall, hKST expression affected body composition only mildly at a very early timepoint and then normalized during the rest of the observational period.

Next, we evaluated glucose metabolism at the age of 12 weeks using an intraperitoneal glucose tolerance test (ipGTT). Glucose and insulin excursion curves did not differ between WT and hKST-TG mice (littermate controls, Figure 2E,F). Also, the gold standard technique to determine insulin sensitivity, the tracer-based hyperinsulinemic euglycemic (HE) clamp, did not indicate differences induced by hKST during NCD (Figure 2G,H). Complete HE clamp parameters including organ-specific glucose uptake, endogenous glucose production and overall glucose uptake are given in Supplementary Table 3.

Additionally, we analyzed possible hKST effects on whole-body energy homeostasis by indirect calorimetry. Energy expenditure (11.69 ± 0.45 kcal/d/mouse in WT vs. 12.23 ± 0.23 kcal/d/mouse in hKST-TG, P = 0.71) and respiratory exchange ratio (0.85 ± 0.016 in WT vs. 0.87 ± 0.015 in hKST-TG, P = 0.98) were similar between genotypes (Supplementary Fig. 1). We also did not detect differences in food and water intake, locomotor activity and VCO_2_ and VO_2_ (Supplementary Table 2).

### 2.3 Effect of hKST on energy homeostasis in DIO mice

**Figure 3:**
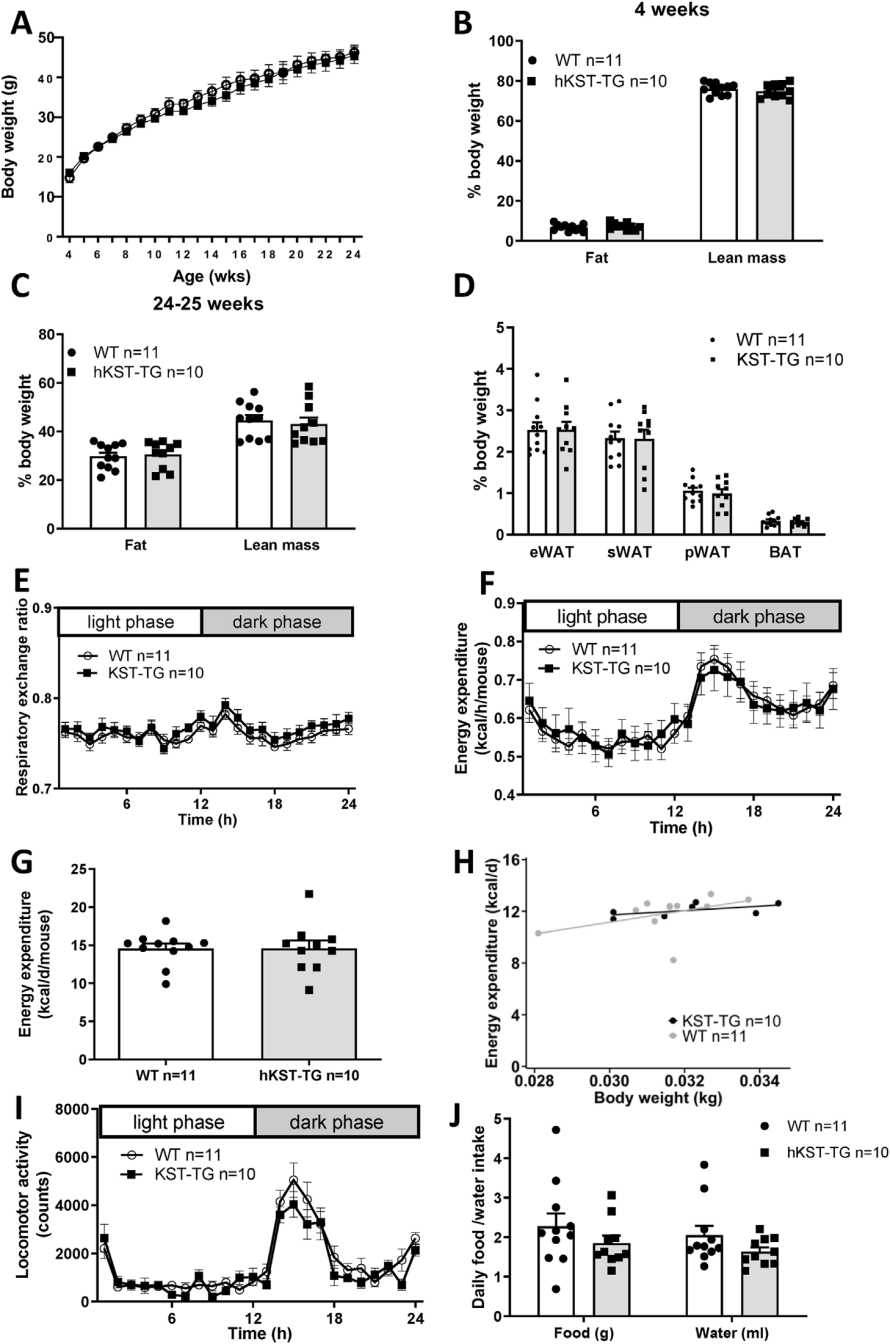
**Body weight and body composition in HFD fed mice.** (A) Development of body weight over 20 weeks. (B) Body fat and (C) lean mass measured by ^1^H nuclear magnetic resonance (NMR) at 4 and 24–25 weeks of age. (D) Weight of eWAT, sWAT, pWAT and BAT. Indirect calorimetry analysis (E) respiratory exchange ratio, (F–H) energy expenditure analyzed over 24 h (F), as mean per day (G) and ANCOVA of daily energy expenditure versus body weight (H), (I) locomotor activity and (J) food/water intake. Data represents mean ± SEM (n = 5–11).

Since hKST did not affect metabolic control in lean, insulin sensitive mice, we next aimed at understanding the role of hKST in metabolic regulation when mice were fed a high-caloric high-fat diet. Therefore, we investigated hKST-TG and WT mice that were fed a HFD for 20 weeks. During the dietary intervention, body weight increased from 14.76 ± 1.18 g to 46.22 ± 1.83 g in WT vs. from 16.48 ± 0.81 g to 46.42 ± 1.77 g in hKST-TG mice (t4 P > 0.99, t24 P > 0.99, Figure 3A). Body fat content increased from 6.69 ± 0.52% to 29.73 ± 1.57% in WT and in hKST from 7.34 ± 0.53% to 29.42 ± 1.78% (t4 P > 0.99, t24 P > 0.99). Lean mass decreased from 76.07 ± 0.91% to 44.53 ± 2.19% in WT and in hKST-TG from 76.11 ± 1.11% to 45.15 ± 2.68% (t4 P > 0.99, t24 P > 0.99, Figure 3B,C). Accordingly, weight of epigonadal, subcutaneous and perirenal white as well as brown adipose tissue depots did not differ between genotypes (Figure 3D). Taken together, under HFD conditions, hKST did not affect body weight or body composition in mice.

Similar to the NCD conditions, we performed indirect calorimetry in 24–25-week-old mice. The respiratory exchange ratio (0.76 ± 0.004 in WT vs. 0.77 ± 0.005 in hKST-TG, P = 0.98, Figure 3E) and energy expenditure (14.58 ± 0.66 kcal/d/mouse in WT, vs. 14.59 ± 1.05 kcal/d/mouse in hKST-TG, P = 0.10, Figure 3F-H) mirrored the dietary condition but revealed no difference between genotypes. Locomotor activity and feeding and drinking behavior were unaltered between WT and hKST-TG mice (Figure 3I,J). The data is summarized in Supplementary Table 2.

### 2.4 Effect of hKST on glucose tolerance and insulin sensitivity in DIO mice

**Figure 4:**
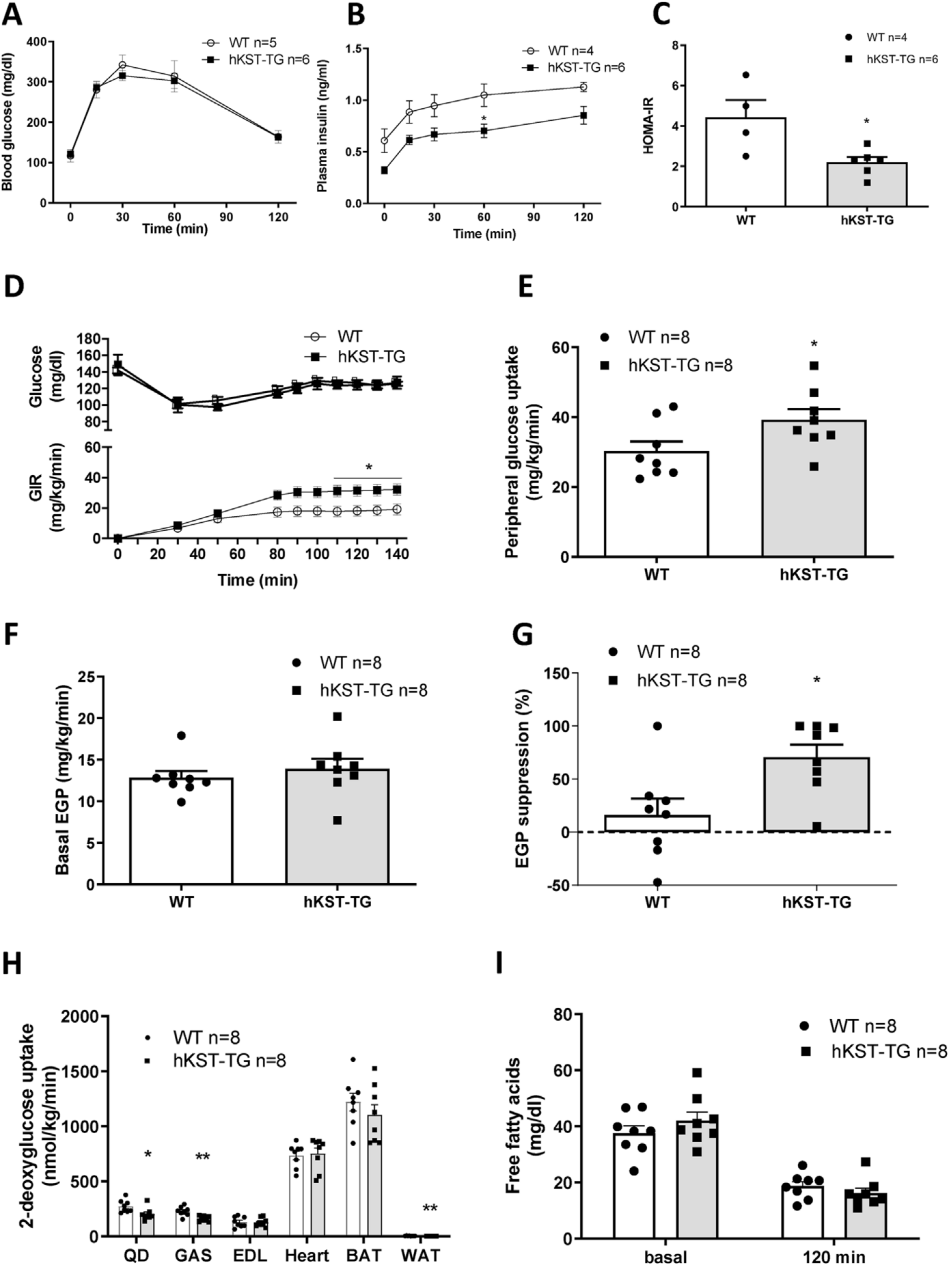
**Glucose metabolism in HFD fed mice.** (A) Blood glucose level and (B) insulin plasma level during ipGTT and consequential (C) calculated HOMA-IR in 12-week-old mice. (D) Glucose infusion rate (GIR), (E) Whole body glucose uptake during steady state, (F) endogenous glucose production rate (EGP) and (G) suppression of EGP during hyperinsulinemic-euglycemic clamp in 26-week-old mice. (H) 2-DG uptake in skeletal muscles (QD, EDL, GAS), heart, WAT and BAT. (I) Free fatty acids during hyperinsulinemic-euglycemic clamp. Data represents mean ± SEM (n = 4–8). ∗P < 0.05, ∗∗P < 0.01.

Next, we performed an ipGTT to determine the effect of hKST on glucose tolerance. Fasting glucose did not differ (116.7 ± 15.17 mg/dl in WT vs. 120.7 ± 21.78 mg/dl in hKST-TG, P = 0.84), fasting insulin was 0.61 ± 0.12 ng/ml in WT vs. 0.32 ± 0.03 ng/ml in hKST-TG, (P = 0.08). During the ipGTT, the glucose excursion curves did not differ between genotypes (Figure 4A), but plasma insulin was markedly reduced in hKST-TG mice during the entire test (Figure 4B), indicating an improved insulin sensitivity. Moreover, the calculated HOMA-IR revealed a significant reduction induced by hKST (Figure 4C). These results suggest that hKST improves diet-induced insulin resistance.

### 2.5 hKST improves hepatic and peripheral insulin resistance in DIO mice

To validate these findings and to better understand the organ-specific contribution to the improvement in insulin sensitivity by hKST, we performed HE clamps in 24–25 week old mice on HFD. Mice were clamped at a blood glucose level of 120 mg/dl and held constant during the steady state phase. Neither body weight nor basal blood glucose was different between genotypes (basal glucose 141.94 ± 3.35 mg/dl in WT vs. 152.09 ± 11.27 mg/dl in hKST-TG, P = 0.41). Glucose infusion rates (GIR) during HE clamps were significantly higher in hKST-TG mice (Figure 4D), further corroborating a beneficial impact of hKST on insulin sensitivity. Peripheral insulin action was evaluated by whole-body ^3^H-labeled glucose uptake and tissue-specific by ^14^C-labeled 2-Deoxy-d-glucose uptake (2-DG). Whole-body glucose uptake in hKST-TG mice was 30% higher compared to WT (39.21 ± 3.10 mg/kg/min in hKST-TG vs. 30.25 ± 2.79 mg/kg/min in WT, P < 0.05, Figure 4E). Basal endogenous glucose production (EGP), mainly deriving from the liver, and insulin-mediated suppression of EGP were determined to evaluate hepatic insulin sensitivity. Whereas basal EGP did not show differences (Figure 4F), insulin-mediated EGP suppression was strongly enhanced in hKST-TG (70.74 ± 11.88% in hKST-TG vs. 25.27 ± 11.71% in WT, P = 0.02), indicating a 3-fold enhanced insulin response in hKST-TG mice compared to WT mice under HFD conditions (Figure 4G). Moreover, whole-body peripheral glucose uptake demonstrated improved overall insulin sensitivity in hKST-TG compared to WT mice on HFD. Rates of glycolysis and glycogen synthesis were similar in both genotypes on both diets (HFD: Supplementary Fig. 4G, data for NCD not shown). Surprisingly, tissue-specific 2-DG uptake in M. gastrocnemius (GAS) and M. quadriceps (QD) indicated reduced uptake, as did epididymal white adipose tissue (eWAT). M. extensor digitorum longus (EDL), heart and BAT did not show differences. Thus, the exact site of enhanced glucose uptake and improved peripheral insulin sensitivity, except for the liver, remains to be determined (Figure 4H). HE clamp data are summarized in Supplementary Table 3. Together, data from ipGTT and HE clamp show improvement in whole-body and hepatic insulin sensitivity, while specific muscle types and white adipose tissue showed reduced glucose uptake.

### 2.6 Effect of hKST on the inflammatory response in DIO

**Figure 5:**
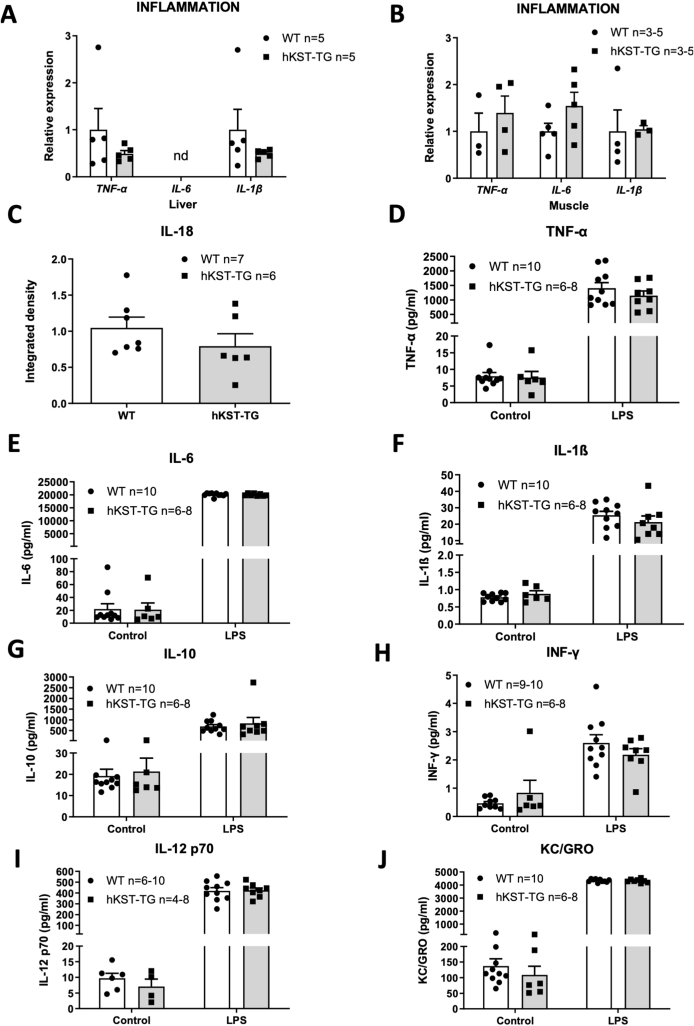
**Inflammatory markers are not different inhKST-TGmice fedaHFD.** (A, B) Measurement of mRNA expression level of inflammatory marker genes in (A) liver and (B) muscle (n = 3). (C) Densitometry analysis of hepatic IL-18 normalized to *β*-Actin, (n = 6–7). (D-J) Changes in inflammatory markers after LPS treatment: Induction of inflammatory markers in plasma after LPS treatment for TNF-*α* (D), IL-6 (E), IL-1β (F), IL-10 (G), INF-γ (H), IL-12 p70 (I), KC/GRO (J). Data represents mean ± SEM (n = 6–10).

We next sought to better understand the mechanisms of improved insulin resistance by hKST. Previous data indicated an anti-inflammatory effect of hKST [17]. Since anti-inflammatory strategies have been shown to effectively improve obesity-associated insulin resistance [[18], [19], [20]], we evaluated the effect of hKST on inflammatory parameters in hKST-TG mice compared to WT littermate controls on HFD. We assessed the expression of major obesity-induced cytokines and inflammatory markers, including tumor necrosis factor-α (TNF-α), interleukin-6 (IL-6), interleukin-1β (IL-1β) in liver and skeletal muscle and interleukin-18 (IL-18) in liver, but observed no differences (Figure 5A,B and C). Since HFD may only be a weak effector of a low-grade meta-inflammation, we hypothesized that the trigger may not have been sufficient to induce pronounced inflammatory effects. To further promote those, hKST-TG and WT littermate control mice on a HFD were injected with bacterial lipopolysaccharides (LPS) or NaCl as control. 2 h after injection, plasma samples were taken and a set of 10 cytokines (IL-1β, IL-2, IL-4, IL-5, IL-6, IL-10, IL-12p70, TNF-α, Interferon-γ, and KC/GRO) was determined (Figure 5D–J). All cytokines increased significantly with LPS treatment (e.g. IL-6 WT w/o 22.08 ± 8.14 pg/ml vs. WT w/LPS 20055.29 ± 210.19 pg/ml, p < 0.001, IL-6 TG w/o 21.14 ± 10.21 pg/ml vs. TG w/LPS 20011.45 ± 154.27 pg/ml p < 0.001). Yet, we did not observe an anti-inflammatory effect of hKST. Taken together, these data suggest that the improvement in insulin sensitivity by hKST is not mediated via an anti-inflammatory action of hKST in this diet-induced obese mouse model.

**Figure 6:**
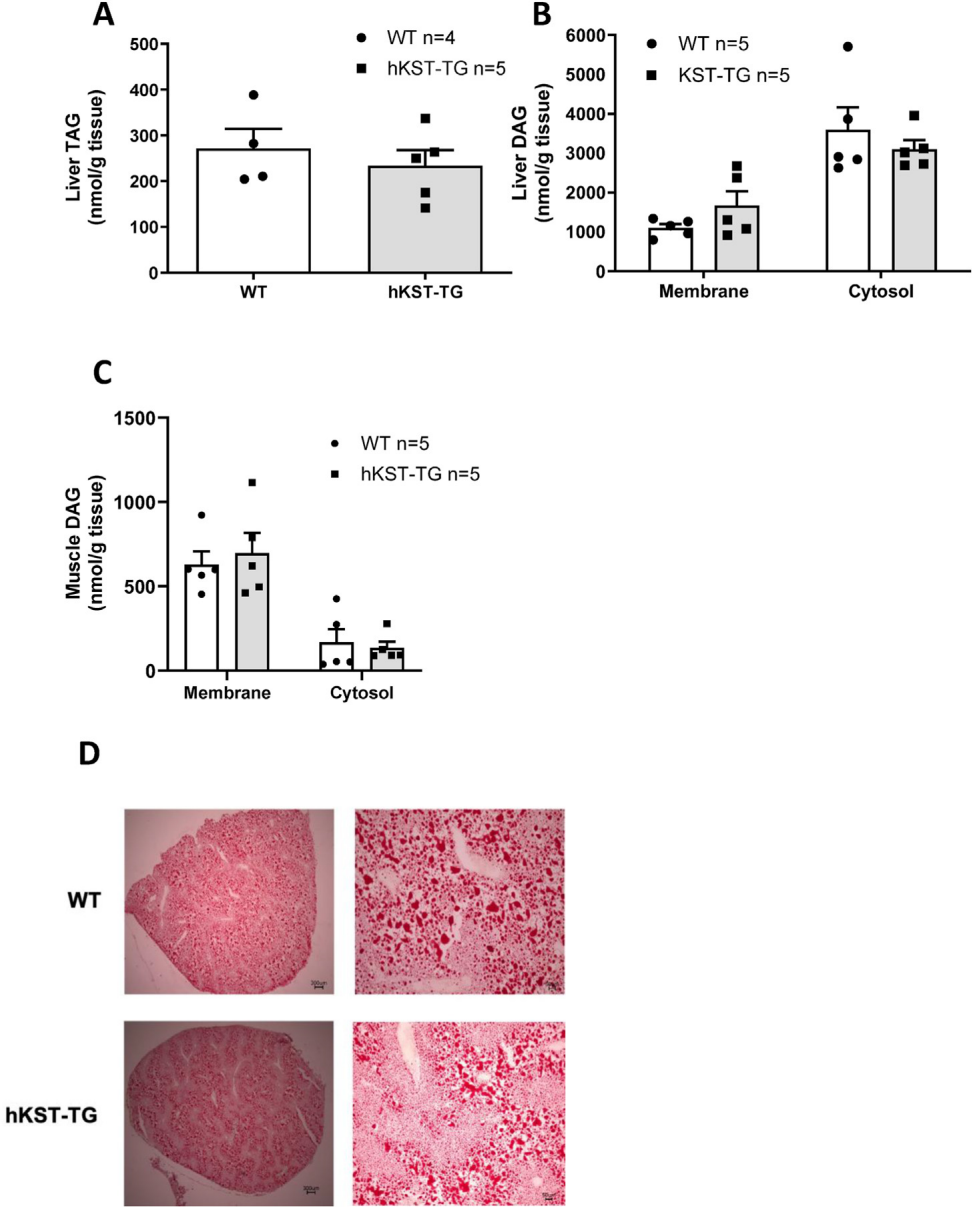
**Hepatic and skeletal muscle lipid content in hKST-TG mice fedaHFD**. (A) Triglycerides (TAGs) in liver lysates (WT n = 4, hKST-TG n = 5). (B-C) Measurement of membrane and cytosolic diacylglycerides (DAGs) in liver (B) and muscle (C, n = 5). (D) Oil-Red-O-stained liver sections (representative pictures in 2× and 10× magnification. Data represents mean ± SEM.

Previous data suggested that hKST may impact ectopic lipid accumulation in the liver via improved adipose tissue insulin sensitivity with consequently reduced lipolysis. Accumulation of ectopic lipids in insulin sensitive organs, including the liver, has also been shown to impact on insulin sensitivity. We, therefore, first determined basal and insulin-suppressed lipolysis before and after the HE clamp. Free fatty acids (FFA) were not different at baseline or after insulin stimulation, ruling out a major effect on lipolysis (Figure 4I). Accordingly, hepatic and skeletal muscle triglycerides (TAGs) and diacylglycerols (DAGs) were determined; the latter lipid species has been shown to mediate diet-induced insulin resistance via the activation of the protein kinase C (PKC) isoforms θ in skeletal muscle and ε in liver [21]. Hepatic lipid and muscle lipid profile of 13 different DAGs in the HFD group (age of 25–26 weeks) were measured. Neither hepatic TAG nor DAG content differed between genotypes, nor skeletal muscle DAGs, and the ratio of membrane to cytosolic DAGs did not differ in liver and skeletal muscle of HFD-fed hKST-TG mice and WT littermate controls (Figure 6A–C). Taken together, neither the previously reported anti-inflammatory response nor a reduction of lipolysis with reduced ectopic lipid accumulation in the absence of changes in body weight explain the effects of hKST on glucose metabolism.

### 2.7 hKST reduces active Wnt/β-catenin signaling in liver

**Figure 7:**
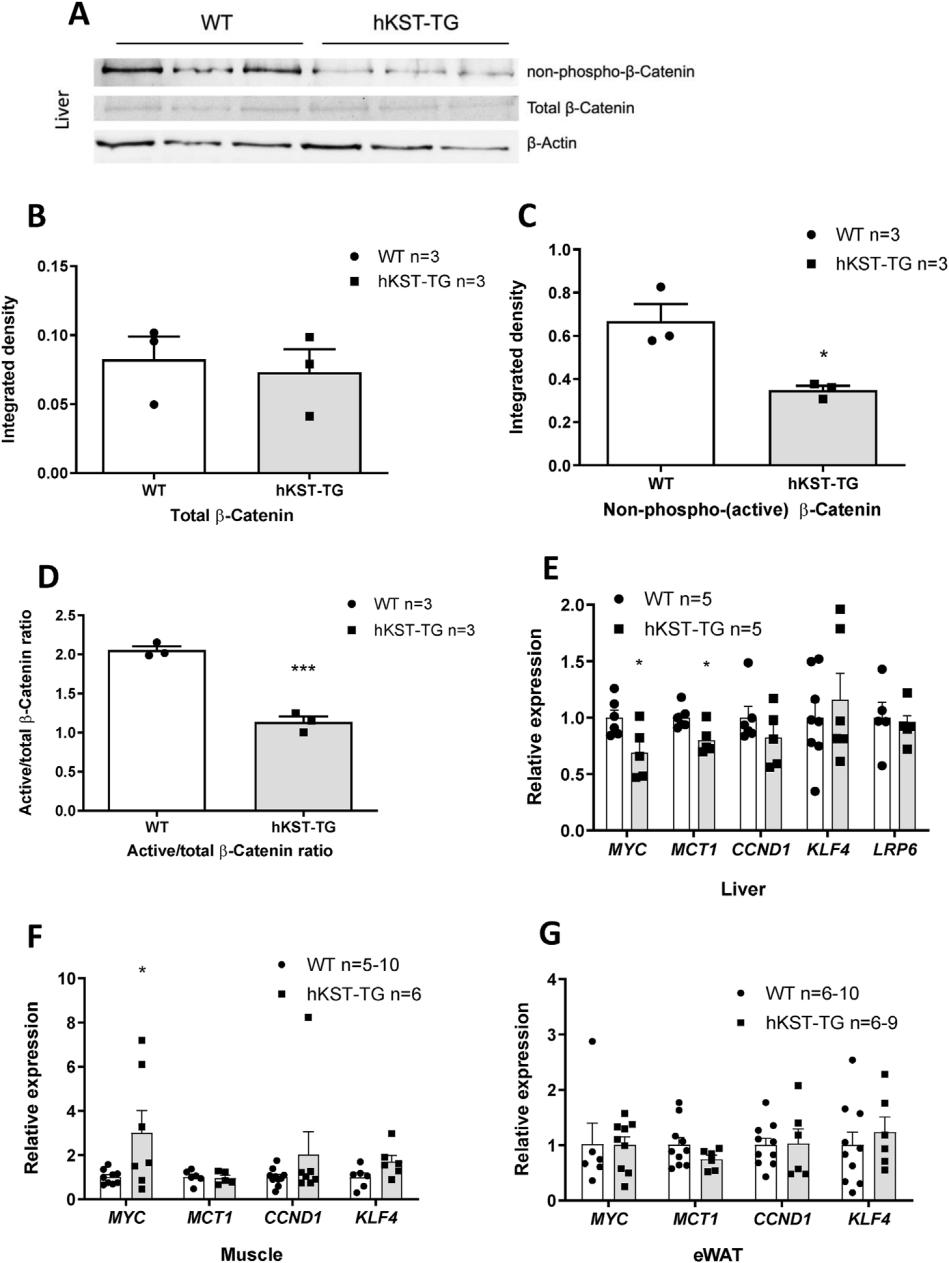
**Wnt/β-catenin pathway in tissues of HFD mice.** (A) Representative Western Blots for non-phospho (active), total β-catenin and β-actin protein abundance in liver of WT and hKST-TG mice (n = 3). (B-D) Densitometry analysis of total β-catenin (B), non-phospho (active) β-catenin (C) and, active/total β-catenin ratio (D) normalized to β-actin. (E–G) Gene expression of Wnt-signaling targets in liver (E), muscle (F) and, eWAT (G). Data represents mean ± SEM (n = 3–10). ∗P < 0.05, ∗∗P < 0.01, ∗∗∗P < 0.001.

To understand the effects of hKST on hepatic glucose metabolism, we next determined active to total β-catenin in livers of hKST-TG and WT mice, since the reduction of the β-catenin pathway co-receptor low-density-lipoprotein-related protein (LRP6) has been shown to improve hepatic insulin sensitivity [22,23]. In hKST-TG mice, hepatic active/total β-catenin was reduced compared to WT (Figure 7A–D). To validate these findings, we determined the known target genes of β-catenin, namely the lactate transporter monocarboxylate transporter 1 (*MCT1*/*SLC16A1*), *c-MYC* and *CYCLIN* *D1*. In line with reduced β-catenin activity, *MCT1* and *c-MYC* exhibited reduced expression in liver of hKST-TG while *CYCLIN* *D1* was only numerically reduced (Figure 7E). Given that insulin sensitivity was not improved in QD and eWAT, Wnt/β-catenin target genes were also analyzed in QD and eWAT as a proxy for Wnt/β-catenin activity. No differences of *MCT1, MYC, KLF4* or *CYCLIN* *D1* were detected in eWAT, whereas *MYC* was strongly upregulated in QD of hKST-TG mice (Figure 7F–G). Given the role of *MYC* in skeletal muscle metabolism, these findings may help explain why insulin sensitivity was not improved or even worsened in QD [24]. In summary, reduced β-catenin signaling in hKST-TG mice may contribute to improved hepatic insulin sensitivity. Clearly, more data are needed to better elucidate the molecular underpinnings of this potential mechanism.

## 3 Discussion

In this study, we show that in patients with overweight and obesity, *KST* mRNA expression in sWAT increased after weight loss. In obese mice, hKST improved hepatic insulin sensitivity, and this effect was independent of body weight, energy expenditure and food intake. In contrast, in the same mouse model, hKST negatively impacted insulin sensitivity in adipose tissue and skeletal muscle. These findings suggest that the role of KST in metabolic regulation is more complex than previous data suggested.

In overweight to obese subjects, weight loss increased *KST* expression in abdominal sWAT. To our knowledge, these data are the first to show that *KST* is expressed in human sWAT and that it increases in this tissue after weight loss on the transcriptional level. Previous data did not observe *KST* expression in human visceral adipose tissue, while the liver expressed hKST at high levels [8]. Moreover, reduced plasma KST levels were found in obese subjects compared to normal weight subjects, and the reduction was independent of hyperglycemia [25], yet a negative association between plasma KST levels and fasting insulin as well as homeostasis assessment model (HOMA-IR), an index of insulin sensitivity, was reported. In contrast, another trial compared patients with obesity with and without prediabetes and showed elevated KST in patients with prediabetes, but not in patients with normal glucose tolerance [26]. KST levels were also decreased in patients with metabolic dysfunction-associated steatotic liver disease (MASLD) compared to patients without fatty liver disease and liver cirrhosis [27,28]. Plasma KST levels were reduced in healthy African-American adolescents with elevated body fat [10], in vitreous fluids of patients with diabetic retinopathy [29], as well as in patients with cardiovascular and other diseases including arterial hypertension, different types of cancer, sepsis and in intestinal biopsies of patients with inflammatory bowel disease [9,11,27,30,31]. The unifying trait of these diseases is the inflammatory nature of the condition going along with oxidative stress. Oxidative stress has been reported to reduce *KST* expression by activating c-Jun NH_2_-terminal kinase-(JNK-)dependent FOXO1 signaling in cultured endothelial cells [32]. Taken together, others and we show that obesity goes along with reduced plasma KST levels and weight loss leads to increased plasma KST levels as well as an increased expression in sWAT.

Given the increase of KST with weight loss, the exact role of KST in metabolic control remained unclear. Studies using adenoviral injection of hKST in epididymal, but not inguinal adipose tissue, mimicking gene therapy, suggested that forced hKST expression reduces body weight in mice fed a 4 week high-fat diet, and remodels adipocyte size and low-grade meta-inflammation [12,13]. While these effects may directly be induced by hKST, weight loss has also persistently been shown to induce such a response in adipose tissue [33]. Thus, it remained unclear if the effect was direct or weight loss-induced. Mechanistic insight into how hKST reduced body weight in these studies remained to be determined. In our model, systemic overexpression of hKST did not induce differences in body weight, energy expenditure, respiratory exchange rate, food intake, drinking volume or locomotor activity between hKST and WT mice, neither on NCD nor on HFD. Interestingly, after 4 weeks on NCD, we observed a moderate numerical difference in body composition with a slight increase in body fat content in hKST mice compared to WT mice. This may indicate adaptive effects that occur at a young age in mice expressing hKST and normalize over time. Thus, studies addressing a period of several months are warranted to appreciate the full picture of hKST effects in metabolic control and previous studies and the duration of our study may be one factor helping to explain the differences found in the phenotypes.

We observed a marked improvement in overall insulin sensitivity in our model. During ipGTT, plasma insulin levels were reduced, while glucose excursion was similar between groups, suggesting improved insulin sensitivity. To further analyze organ-specific contributions to hKST-mediated improved insulin sensitivity, we performed HE clamps. We observed improved overall insulin sensitivity as reflected by a higher glucose infusion rate in hKST-TG mice. Regulation of EGP accounted for the improvement, since addition of insulin during the clamp suppressed EGP more than 4-fold in hKST-TG mice compared to WT littermate controls. Moreover, we observed a slight increase in overall peripheral glucose uptake in hKST-TG mice. The finding that glucose uptake was rather reduced in some skeletal muscle types and WAT is novel, has important implications and deserves further study. It may suggest that hKST induces a complex interaction between liver, WAT and skeletal muscle, for example by secreted factors from the liver, but clearly, this notion needs further prove. The overall insulin sensitizing effect of hKST however is an important and relevant (patho)physiological hKST action in the setting of DIO and adds to the pleiotropic functions of the protein, including vasodilation as well as inhibition of angiogenesis, apoptosis, fibrosis, tumor growth, and metastasis in rodents [[34], [35], [36]]. Yet, reducing skeletal muscle and adipose tissue glucose uptake needs to be considered if hKST was to be developed further into a therapeutic target for metabolic disease.

Several studies indicated that KST exerts anti-inflammatory effects by interfering with cell surface heparan sulfate proteoglycans and thereby antagonizing the signaling pathways mediated by VEGF, TNF-α, TGF-β, and Wnt [10,11,28,37]. Along these lines, KST has been reported to inhibit LPS- and TNF-α-induced inflammation and TNF-α-mediated lipid peroxidation in human adipocytes [8,17,25,38]. Obesity is characterized by a chronic inflammatory state [34,39,40]. The nature of the chronic immune response is unique compared to an acute inflammatory response. Accumulation of immune cells such as macrophages and T-lymphocytes in expanded adipose tissue and the liver leads to an elevated secretion of inflammatory cytokines, interfering with the insulin signaling cascade and Acetyl-CoA levels in the liver. Together, these actions may contribute to insulin resistance induced by obesity [18,39,41,42]. Yet, we did not observe effects of hKST on the expression of inflammation markers (*TNF-α*, *IL-6, IL-1β*, *IL-18*) in liver or skeletal muscle or responses of ten selected cytokines in plasma following LPS stimulation. These findings rule out that the effect of KST on insulin sensitivity in our study was mediated by its anti-inflammatory properties. Differences to other studies may again be explained by differences in body weight.

Disproportional and/or ectopic lipid accumulation is another mechanism contributing to insulin resistance in the setting of DIO [21,43]. Accordingly, ectopic lipid accumulation in the liver and in skeletal muscle develops when lipid supply or lipogenesis overrides fatty acid β-oxidation or lipid export from the liver. Specific intermediates of TAG metabolism, DAGs, induce PKCε in the liver [44,45] and PKC-θ in skeletal muscle [46], inhibiting insulin receptors by serine phosphorylation. However, we did not detect changes in adipose tissue depot size, hepatic or skeletal muscle TAG or DAG content. Thus, the insulin sensitizing effect of hKST in our model cannot simply be attributed to changed ectopic lipid accumulation. Also, adipose tissue lipolysis was not different in our mouse model between the genotypes. Again, differences in body weight may help to explain differences to another model.

KST is a serine proteinase inhibitor [38,47] and functions as an endogenous antagonist of LRP6 and inhibitor of Wnt signaling [48]. The Wnt/β-Catenin signaling pathway mediates cell proliferation, differentiation and migration [49]. Wnt signaling is a tightly regulated pathway comprised of Wnt ligands, frizzled (FZD) receptors, and co-receptors, including LRP5/6, an intracellular signaling molecule cascade and the effector β-catenin [50]. Non-phosphorylated β-catenin plays an essential role in the canonical Wnt pathway (or Wnt/β-catenin pathway). Recent genome-wide association studies suggested a link between the Wnt pathway and metabolic diseases [[51], [52], [53]] and studies in humans and rodents have identified a mutation in LRPp6 that predisposes to the metabolic syndrome [54,55]. In rodents, second generation antisense oligonucleotides against β-catenin improved hepatic insulin sensitivity and insulin-stimulated whole-body glucose metabolism [23]. We, therefore, assessed active, non-phosphorylated β-catenin as well as total β-catenin in the liver of DIO mice. We observed a reduced ratio of active/total β-catenin and a reduction of Wnt signaling target genes *MCT1* and *MYC* [[56], [57], [58]] in the liver, but not in adipose tissue and skeletal muscle. In line with this observation, insulin-mediated suppression of hepatic glucose production was reduced in hKST-TG mice, but glucose uptake into adipose tissue and skeletal muscle was not higher under HE clamp conditions. Interestingly, partial deletion of *MCT1*, a major lactate transporter of the solute carrier family 16 (SLC16), has been shown to protect from DIO-associated metabolic perturbances [[59], [60], [61]], and *c-MYC* overexpression has been linked to diabetes development in transgenic mice [62]. It is tempting to speculate that via the reduction of MCT1, also other pleiotropic actions of hKST may be at least in part mediated [63]. Together, these data may indicate that hKST can regulate the Wnt/β-catenin pathway in a tissue-dependent manner. The differential regulation was also observed for retina and skin [2,11]. This is underlined by our finding that Wnt/β-catenin in the liver was affected by hKST, where insulin sensitivity was improved, whereas in skeletal muscle and WAT, where Wnt/β-catenin signaling did not seem to be affected by hKST, insulin resistance was unchanged or even reduced. Still, other pathways could also play a role in mediating effects of KST in metabolism. Yet, we excluded major differences in genes involved in adipogenesis and angiogenesis amongst others (Supplementary Figs. 4–5). Moreover, while effects of KST on protein kinase B (AKT), FOXO1 and peroxisome proliferator activated receptor γ (PPARγ) have been described [4,64], we did not observe differences in these pathways in our model. In another mouse model, visceral adipose tissue-derived exosomes that ameliorated obesity-induced hepatic insulin resistance were described [12], which may be less important in our model with systemic overexpression of hKST.

Furthermore, inhibition of proteases could also contribute to an improved glucose regulation by KST. KLK7 leads to insulin degradation in murine pancreatic islets [65]. By inhibiting KLK7 [5], KST could improve glucose regulation. However, we do not suspect this to be the driving mechanism in our study since insulin levels were reduced due to improved insulin sensitivity, and in the NCD mice, without a difference in insulin sensitivity, we did not observe a reduction in insulin levels.

Our study has several limitations. First, we measured only selected inflammatory markers. Second, we exclusively analyzed male mice to exclude confounding factors induced by the menstrual cycle, which have been shown to affect insulin sensitivity [66,67]. Furthermore, SERPINA3C, which has also been shown to counteract adipose inflammation under HFD [68,69], and is considered as the murine homolog of human SERPINA4, was used to characterize endogenous *KST* expression, not revealing a difference between genotypes. SERPINA3C shows a higher degree of homology with SERPINA3 (antichymotrypsin, 59%) than with SERPINA4 (45%), which still represents a robust homology [69,70]. Moreover, [2–^3^H]-D-glucose was used in the HE clamp, which may lead to an overestimation of glucose turnover [71]. Finally, KST expression in our mouse model did most likely not reflect the physiological KST organ distribution. Potentially, the KST expression pattern may have negatively affected skeletal muscle insulin sensitivity, which might not be present in a more physiological setting. Physiologically, KST is mainly produced by the liver [38]. Therefore, a liver-specific hKST expression model would be of special interest for future research.

## 3.1 Summary

As obesity and T2D are major health threats worldwide, a more detailed understanding of the pathophysiology and potential therapeutic targets is needed, also because current therapeutic strategies are not sustainable beyond the active treatment period. hKST has been suggested to be such a potential target due to its pleiotropic actions. We show that human KST improved diet-induced insulin resistance in mice, without changing body composition or energy metabolism. The improvement may be explained by the activation of Wnt/β-catenin signaling with subsequent induction of specific target genes in the liver. Given the fact that we also observed that weight loss in humans with obesity leads to increased KST levels, it seems possible that KST is a contributing factor to the improvement in insulin sensitivity after weight loss. Yet, our data also indicate that hKST may differentially regulate insulin sensitivity in adipose tissue and skeletal muscle, which needs to be kept in mind for future developments of hKST as a therapeutic tool. Our findings add to the plethora of beneficial and complex actions of KST, such as blood pressure control, regulation of fibrosis and tumor growth. Thus, KST may be an exciting, yet challenging, therapeutic target for patients with metabolic syndrome.

## 4 Research design and methods

### 4.1 Human subjects

Human subcutaneous fat cDNA samples were obtained from the clinical trial “Comparison of Low Fat and Low Carbohydrate Diets With Respect to Weight Loss and Metabolic Effects (B-SMART)” (ClinicalTrials.gov Identifier: NCT00956566). Clinical data were kindly provided by Dr. Sven Haufe and his team (Hannover Medical School) in an anonymous and pseudonymous manner. We correlated body fat mass and HbA1c with *KST* mRNA expression in sWAT as a post-hoc analysis from the B-SMART study.

The B-SMART study is a prospective, randomized weight reduction study. Patients (overweight to obese healthy women and men) received a hypocaloric diet reduced in carbohydrates or fat over a period of 6 months. The energy was restricted to −30% of energy intake before diet (to a minimum of 1200 kcal/day) to ensure significant weight reduction. The primary aim of the study was to evaluate if low carbohydrate hypocaloric diets are similarly effective compared to fat reduced hypocaloric diets in ameliorating obesity-related hepatic steatosis. After the dietary intervention, weight reduction and associated metabolic and cardiovascular markers were compared.

### 4.2 Animal care and studies

Animal care and procedures were performed in accordance with the guidelines of the Charité - Universitätsmedizin Berlin. All experiments were approved by the Landesamt für Gesundheit und Soziales (LAGeSo, Berlin, Germany) for the use of laboratory animals and are in accordance with the current version of the German Law on the Protection of Animals. hKST-TG mice were housed in groups under specific pathogen free (SPF) condition in individually ventilated cages (IVC). All mice were maintained on 12 h light–dark cycle with ad libitum access to water and food. Mice were bred in order to achieve hKST-TG mice or wild type littermate control mice. After genotyping, male mice were fed a normal-chow (R/M−H V1534-300, Ssniff, Soest, Germany) or high-fat diet (60% kcal from fat, D12492 (I), Ssniff, Soest, Germany) starting at an age of 4 weeks for 20–22 weeks. Body weight was assessed every week.

#### 4.2.1 Generation of Kallistatin transgenic mice

Transgenic mice were kindly provided by the group of Jian-Xing Ma from the University of Oklahoma, USA. Systemic overexpression of human KST is driven by a chicken β-Actin promotor and cloned into the pTriEx1.1 vector (Novagen, Darmstadt, Germany) as described previously [11]. Expression level and plasma concentration of murine and human KST are stated in Supplementary Fig. 2.

#### 4.2.2 Body composition and indirect calorimetry

Body fat and lean mass were assessed at different time points via ^1^H-nuclear magnetic resonance (NMR) spectroscopy using a Minispec LF50 Body Composition Analyzer (Bruker BioSpin, USA).

For metabolic characterization, mice were individually housed for a period of 60 h in the PhenoMaster Cage System (TSE, Systems GmbH, Bad Homburg, Germany). After an adaption time of 12 h, a 48 h measuring period followed and oxygen consumption, carbon dioxide production, food and water consumption as well as physical activity were monitored.

Calculation of respiratory exchange ratio and energy expenditure (EE) were done after analysis. Caloric intake was adjusted for lean body mass.

#### 4.2.3 Intraperitoneal glucose tolerance test (ipGTT)

ipGTTs were performed in 12-week-old mice in the morning at 8 am. For the test, mice were single-caged and fasted overnight. For blood sampling, mice were put into a restrainer and blood glucose was measured using automated glucometers (Contour® XT and Contour® next sensors, Bayer Ascensia Diabetes Care, Leverkusen, Germany). Blood samples from the tail were taken via micro hematocrit tubes (Brand, Wertheim, Germany). Tail blood was collected at time point 0, 15, 30, 60 and 120 min following intraperitoneal injection of 1 mg/g body weight of glucose (10%). Blood samples were immediately stored on ice. Plasma was obtained after 10 min centrifugation, at 10,000 rpm, 4°C and stored at −80°C.

The homeostasis assessment model (HOMA-IR) was calculated using a mathematic equation including fasting glucose and insulin level (HOMA IR = fasting insulin level (mU/l) ∗ fasting glucose level (mg/dl)/405).

#### 4.2.4 Hyperinsulinemic-euglycemic (HE) clamp

All HE clamp studies were performed according to protocols published previously [72,73]. In brief, mice received an implantation of a jugular venous catheter under isoflurane anesthesia. After a 7 days recovery period in single-housing, mice were fasted 16 h overnight. In the morning, body weight was determined and mice were placed in restrainers. Tail tip was cut for blood sampling, first blood sample was taken and catheters were connected to infusion syringes located in microdialysis pumps. To assess basal glucose turnover, [2–^3^H]-d-glucose (Hartmann Analytic, Braunschweig, Germany) was infused at 0.05 μCi/min for 120 min. HE clamp started with a 3 min priming infusion (0.25 μCi/min) and a subsequent 140 min continuous infusion (0.1 μCi/min) of [2–^3^H]-D-glucose and 3 mU/kg/min human insulin (Insuman rapid, Sanofi-Aventis, Frankfurt, Germany). Simultaneously, a variable infusion of 20% glucose (B. Braun, Melsungen, Germany) was started from time point 0 to maintain euglycemia at 120 mg/dl. After 85 min, 10 μCi 2-[1–^14^C]-deoxy-D-glucose(Hartmann Analytic, Braunschweig, Germany) was injected as bolus to elucidate organ-specific glucose uptake. Blood was collected via heparinized capillaries at time point 0, and every 10 min from time point 90. Blood glucose levels were measured in duplicates with handheld glucometers and matching test stripes (Glucometer Contour® XT and Contour® next sensors, Bayer Ascensia Diabetes Care, Leverkusen, Germany), at −5, 30, 50, 65, 80, 90, 100, 110, 120, 130 and 140 min. At 140 min, mice were anesthetized with Ketamin/Xylazin injection (100/12 mg/kg). Tissues were quickly excised, compressed snap-frozen in liquid nitrogen and stored at −80°C for further analyses. To compensate volume loss through blood sampling during insulin-stimulated period, mice received an albumin-containing solution (4.2 μl/min) mimicking artificial plasma together with the continuous infusion [66]. Immediately after sampling, blood samples were centrifuged for 1 min at 13,000 rpm at 4°C and plasma was transferred to fresh tubes. For tracer analysis, 10 μl plasma was diluted with 20 μl distilled water. 30 μl [2–^3^H]-D-glucose (F1) as well as 30 μl [2–^3^H]-D-glucose mixed with insulin (F2) pre and post infusion were diluted with 60 μl distilled water. Plasma samples were deproteinized with ZnSO_4_ and Ba(OH)_2_. One part of the sample was counted directly as non-dried sample in scintillation fluid (Insta-Gel Plus, PerkinElmer, Waltham, USA) to determine total ^3^H content (non-metabolized glucose and ^3^H_2_O derived from ^3^H glucose). The other part was dried to remove ^3^H_2_O, resuspended in H_2_O and counted in scintillation fluid to determine the non-metabolized glucose. The difference (non-dried – dried) is determined as metabolized glucose and calculated to the steady state glucose measured with the glucometers.

### 4.3 Tissue-specific glucose uptake

Tissue samples were homogenized by ultra-turrax and boiled for 10 min at 95°C in a water bath. The supernatant was transferred in anion exchange columns (Poly-Prep Prefilled Chromatography Columns, BioRad, Hercules, USA) to separate 2[^14^C]-deoxy-DG6P from 2[1–^14^C]-deoxy-DG, following three washing steps with 2 ml distilled water. Wash fractions were collected in one tube per sample. The elution of the metabolized 2-[^14^C]-deoxy-DG6P was done with three times by adding 2 ml of 0.2 M formic acid/0.5 M ammonium acetate solution. Formic acid is more negatively charged compared to phosphate and displaces the 2-[^14^C]-deoxy-DG6P in the column. 40 μl of supernatant mixed with 460 μl distilled water, 500 μl of the eluate and 500 μl of wash fraction were vortexed with 3 ml scintillation solution and measured for 10 min. The eluate contains the glucose that was taken up and metabolized by the cells of the respective tissue. Calculations were done according to [74,75].

### 4.4 Administration of lipopolysaccharides (LPS)

LPS from E. coli, serotype R515 (Re), was purchased from Enzo (Lausen, Switzerland). For the LPS-challenged *in vivo* experiment, 20-week-old mice fed a HFD were injected intraperitoneally with LPS (0.25 mg/kg) or 0.9% NaCl (200 μl). After 2 h incubation time, mice were anesthetized with isoflurane and blood was collected via the intracardial route. Blood samples were immediately stored on ice and plasma was obtained after 10 min centrifugation with 10,000 rpm at 4°C and stored at −80°C.

### 4.5 Oil-Red-O staining

Oil-Red-O staining was performed as described previously [76]. Liver tissue was fixed in 4% paraformaldehyde, dehydrated in 20% sucrose solution and subsequently embedded in TissueTek (Sakura Finetek, Umkirch, Germany) for cryostat sectioning. 15 μM-thick liver sections were stained using Oil-Red-O solution (Sigma–Aldrich, Missouri, USA, 3 mg/ml) and microscopy was performed at the Axio Observer Z.1 widefield microscope using Zen 2.3 software (Carl Zeiss microscopy, Oberkochen, Germany).

### 4.6 Biochemical analysis

Plasma insulin levels were measured using mouse/rat insulin ELISA (Crystal Chem., Downers Grove, USA). Plasma FFAs were measured by NEFA-HR(2) Assay (WAKO Chemicals, Neuss, Germany). Liver and muscle DAGs were measured by liquid chromatography-mass spectrometry/mass spectrometry in the lab of Gerald I. Shulman (Yale University, USA) as described previously [77]. Hepatic TAG content was determined using a triglyceride assay (DiaSys, Holzheim, Germany) following the manufacturer's instructions. Inflammation markers in plasma samples were analyzed by electrochemiluminescence assays (Meso Scale Discovery, Rockville, USA).

### 4.7 Western blot

Equal amounts of proteins (20–30 μg) were diluted in 4× SDS buffer (8% SDS, 10% β-mercaptoethanol, 40% glycerol, 0.008% bromophenol blue and 250 mM Tris, pH 6.8) and boiled at 95°C for 5 min prior to sodium dodecyl sulfate-polyacrylamide gel electrophoresis (SDS-PAGE). For the separation, self-casted 10% SDS-gels were used. The blotting was performed in Wet/Tank blotting system (Bio-Rad, Munich, Germany) according to the manufacturer's protocol. Nitrocellulose membranes (Roth, Karlsruhe, Germany) were used and transfer was done at 100 V. All antibodies were purchased form Cell Signaling Technologies (Danvers, USA) and used as recommended (1:1000). Immune complexes were detected using enhanced chemiluminescence reagent (Pierce™ ECL Plus Western Blotting Substrate, Thermo Fisher Scientific, Schwerte, Germany). Imaging and quantification were performed with BioSpectrum Imaging System (Bio-Rad).

### 4.8 mRNA expression analysis

RNA was extracted with TRIzol® Reagent (Thermo Fisher Scientific, Schwerte, Germany) based on the manufacturer's protocol. MRNA expression was assessed by real-time PCR on MX 3000 (Stratagene, La Jolla, USA) with a *SYBR Green* detection system. Samples were measured in triplicates and calculations were performed using a comparative method (2^−ΔΔCt^).

Liver and fat data were normalized to *ß-Actin, HPRT-1* or *POLR2A* and muscle to *HPRT-1*. Primer sequences have been previously described [76,78] and are listed in Supplementary Table 4.

### 4.9 Statistics

Data are expressed as means ± standard error of the mean (SEM). All statistical analyses were assessed using a two-tailed t-test or two-way analysis of variance with multiple comparison and Bonferroni post hoc test after normal distribution was evaluated (GraphPad Prism 10, La Jolla, USA).

## Funding

The study was supported by a grant from the German Research Association to Andreas L. Birkenfeld (BI1292/11-1, BI1292/13-1, GRK 2812), as well as funding via the German Center for Diabetes Research (DZD, 01GI0925) via the German Federal Ministry of Education and Research. Jian-Xing Ma is the recipient of NIH grants EY033330 and EY033477, measurement of the DAGs by Gerald I Shulman was supported by NIH/NIDDK grant P30DK045735.

## CRediT authorship contribution statement

**Leontine Sandforth:** Writing – review & editing, Writing – original draft, Visualization, Validation, Methodology, Formal analysis, Conceptualization. **Sebastian Brachs:** Writing – review & editing, Validation, Methodology, Data curation. **Julia Reinke:** Writing – original draft, Visualization, Methodology, Formal analysis, Conceptualization. **Diana Willmes:** Writing – review & editing, Methodology, Formal analysis. **Gencer Sancar:** Methodology, Formal analysis. **Judith Seigner:** Methodology, Formal analysis. **David Juarez Lopez:** Methodology, Formal analysis. **Arvid Sandforth:** Writing – review & editing, Data curation. **Jeffrey D. McBride:** Writing – review & editing, Methodology, Conceptualization. **Jian-Xing Ma:** Writing – review & editing, Methodology, Conceptualization. **Sven Haufe:** Writing – review & editing, Validation, Methodology, Formal analysis, Data curation. **Jens Jordan:** Writing – review & editing, Methodology, Conceptualization. **Andreas L. Birkenfeld:** Writing – review & editing, Writing – original draft, Supervision, Funding acquisition, Conceptualization.

## Declaration of competing interest

Jens Jordan receives grants from Novo-Nordisk, Boehringer-Ingelheim, NASA and ESA as well as honoraria from Menarini and Berlin Chemie. He participates in boards of Novo-Nordisk, Theravance, ESH, EFAS and DGLRM and is the co-founder of Eternygen GmbH. All other authors declare that they have no known competing financial interests or personal relationships that could have appeared to influence the work reported in this paper.

## Data Availability

Data will be made available on request.
