# Supplementary material for: Role of human Kallistatin in glucose and energy homeostasis in mice
Source: Mol Metab. 2024 Feb 29;82:101905. doi: 10.1016/j.molmet.2024.101905 (PMC10937158; doi:10.1016/j.molmet.2024.101905)
Supplement: Multimedia component 1 [file mmc1.pptx]

## Slide 1
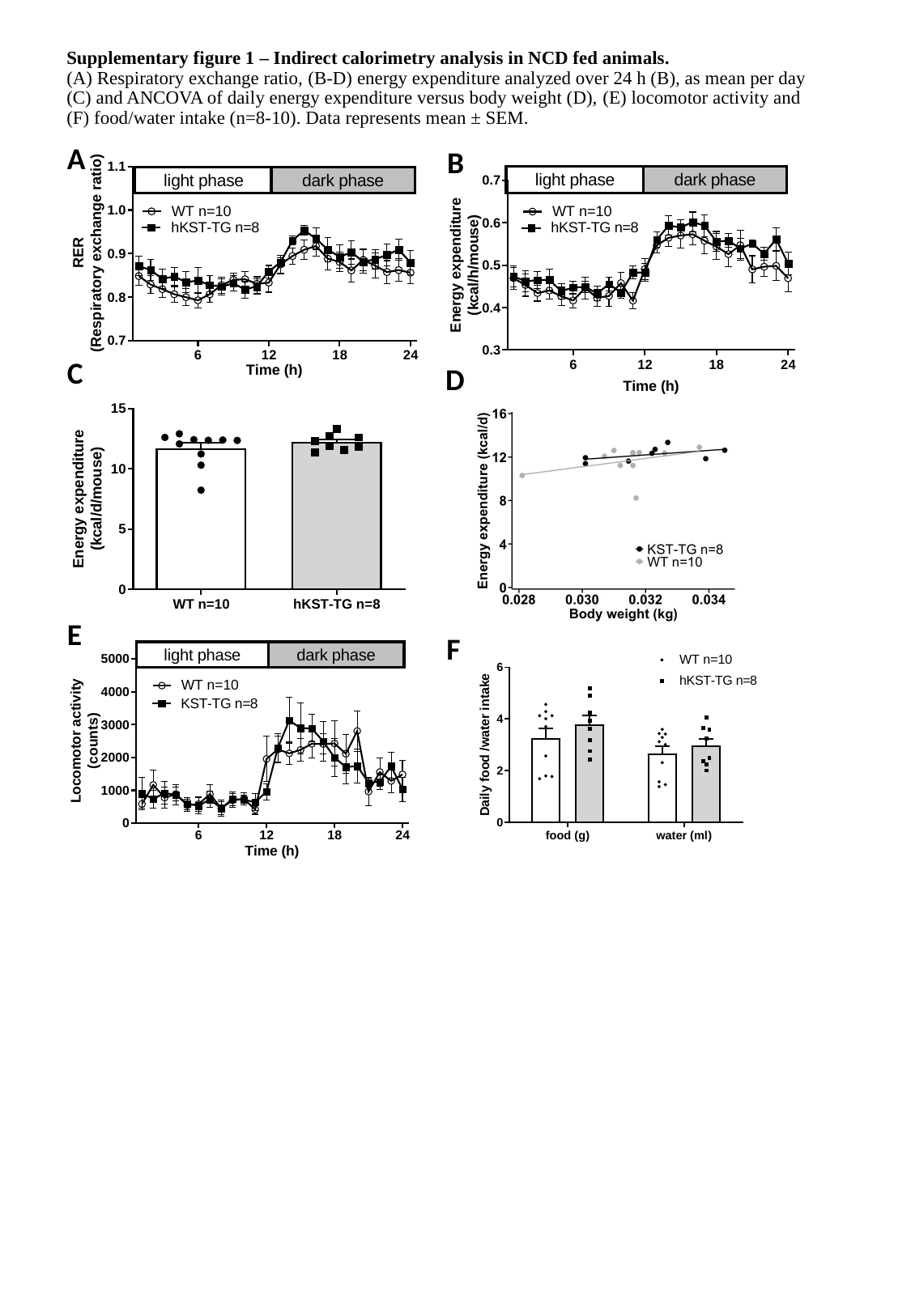

Supplementary figure 1 – Indirect calorimetry analysis in NCD fed animals.
(A) Respiratory exchange ratio, (B-D) energy expenditure analyzed over 24 h (B), as mean per day (C) and ANCOVA of daily energy expenditure versus body weight (D), (E) locomotor activity and (F) food/water intake (n=8-10). Data represents mean ± SEM.
A
B
C
D
E
F

## Slide 2
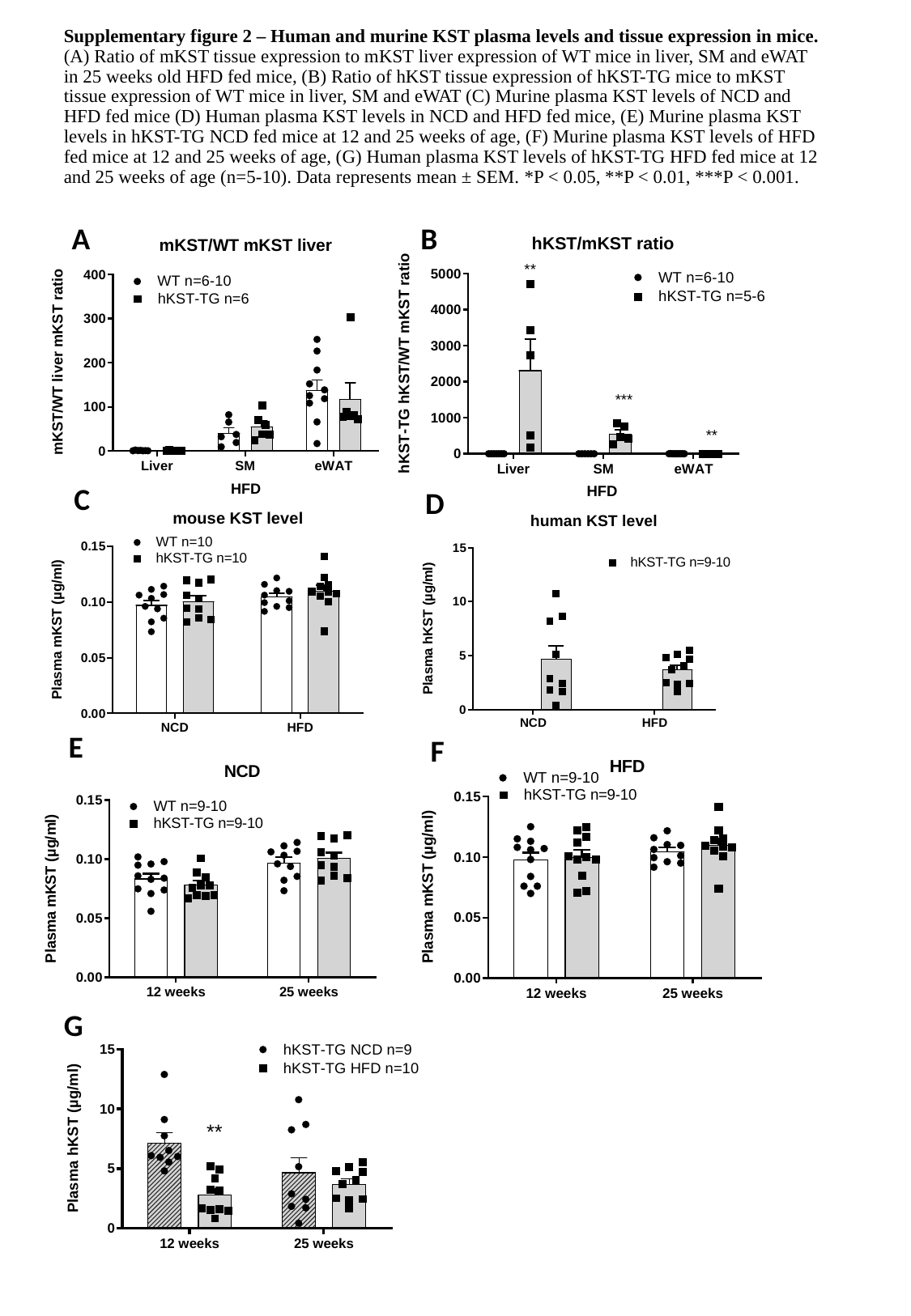

Supplementary figure 2 – Human and murine KST plasma levels and tissue expression in mice.
(A) Ratio of mKST tissue expression to mKST liver expression of WT mice in liver, SM and eWAT in 25 weeks old HFD fed mice, (B) Ratio of hKST tissue expression of hKST-TG mice to mKST tissue expression of WT mice in liver, SM and eWAT (C) Murine plasma KST levels of NCD and HFD fed mice (D) Human plasma KST levels in NCD and HFD fed mice, (E) Murine plasma KST levels in hKST-TG NCD fed mice at 12 and 25 weeks of age, (F) Murine plasma KST levels of HFD fed mice at 12 and 25 weeks of age, (G) Human plasma KST levels of hKST-TG HFD fed mice at 12 and 25 weeks of age (n=5-10). Data represents mean ± SEM. *P < 0.05, **P < 0.01, ***P < 0.001.
A
B
C
D
E
F
G

## Slide 3
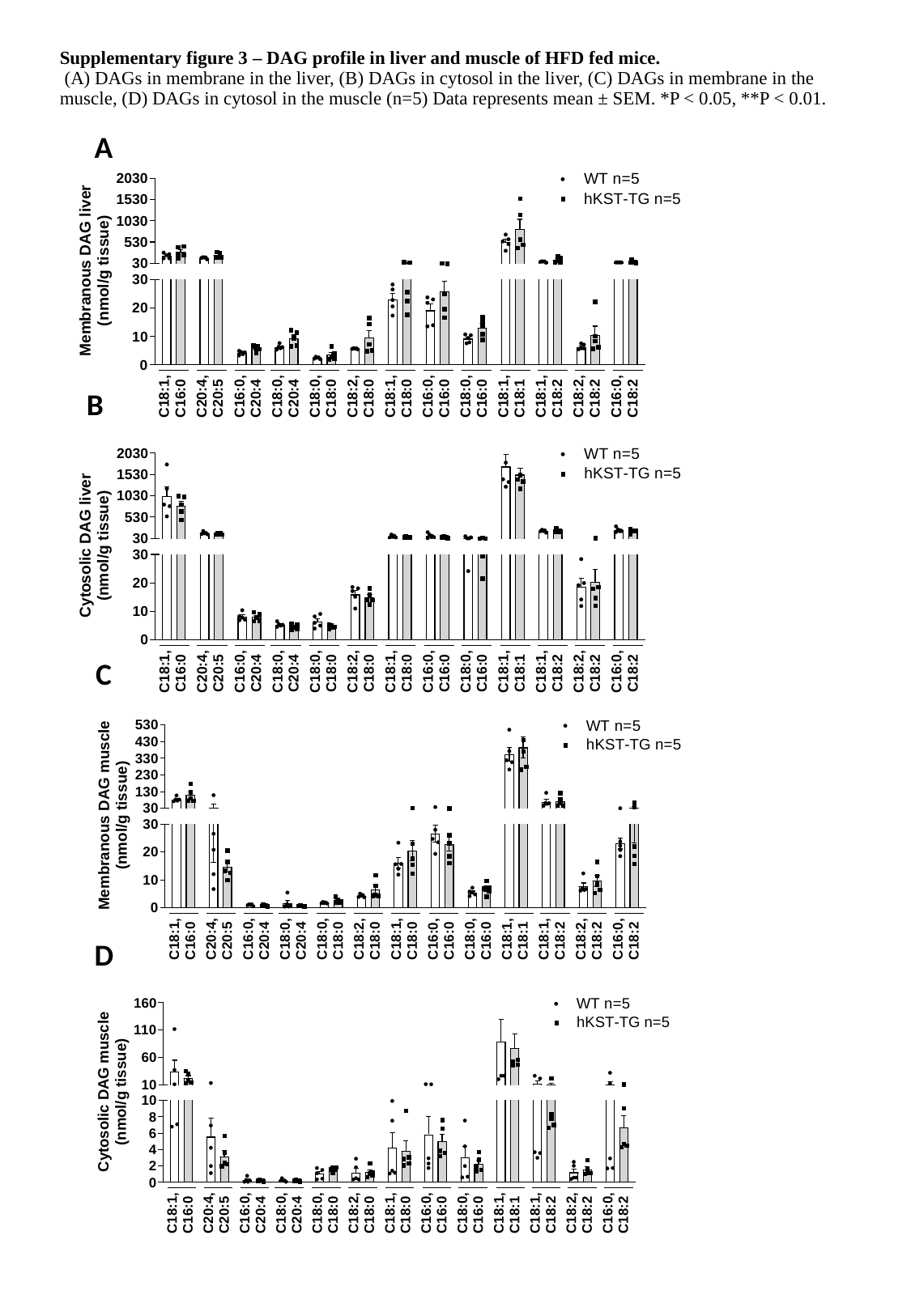

# Supplementary figure 3 – DAG profile in liver and muscle of HFD fed mice. (A) DAGs in membrane in the liver, (B) DAGs in cytosol in the liver, (C) DAGs in membrane in the muscle, (D) DAGs in cytosol in the muscle (n=5) Data represents mean ± SEM. *P < 0.05, **P < 0.01.
A
B
C
D

## Slide 4
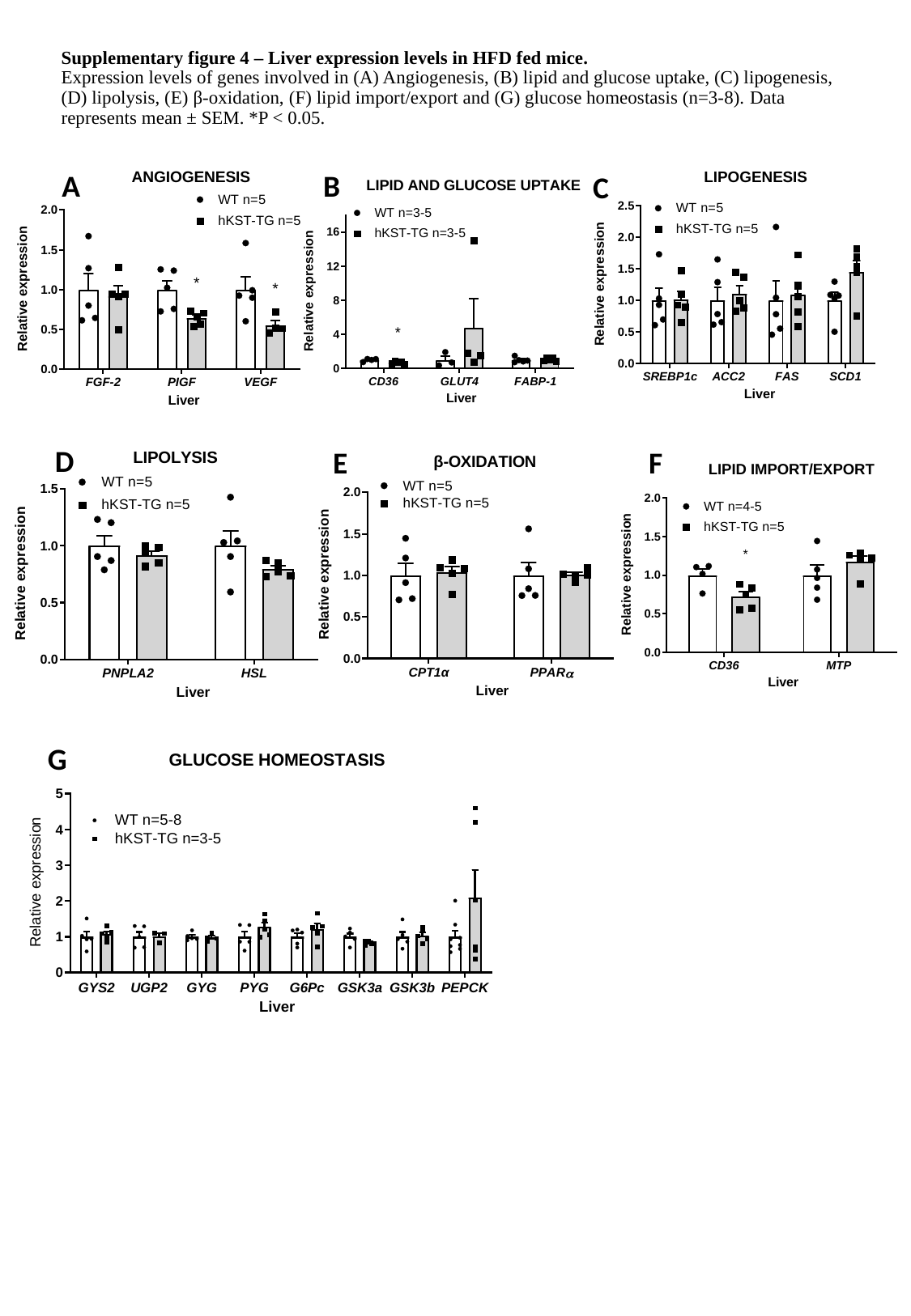

Supplementary figure 4 – Liver expression levels in HFD fed mice.
Expression levels of genes involved in (A) Angiogenesis, (B) lipid and glucose uptake, (C) lipogenesis, (D) lipolysis, (E) β-oxidation, (F) lipid import/export and (G) glucose homeostasis (n=3-8). Data represents mean ± SEM. *P < 0.05.
A
B
C
D
E
F
G

## Slide 5
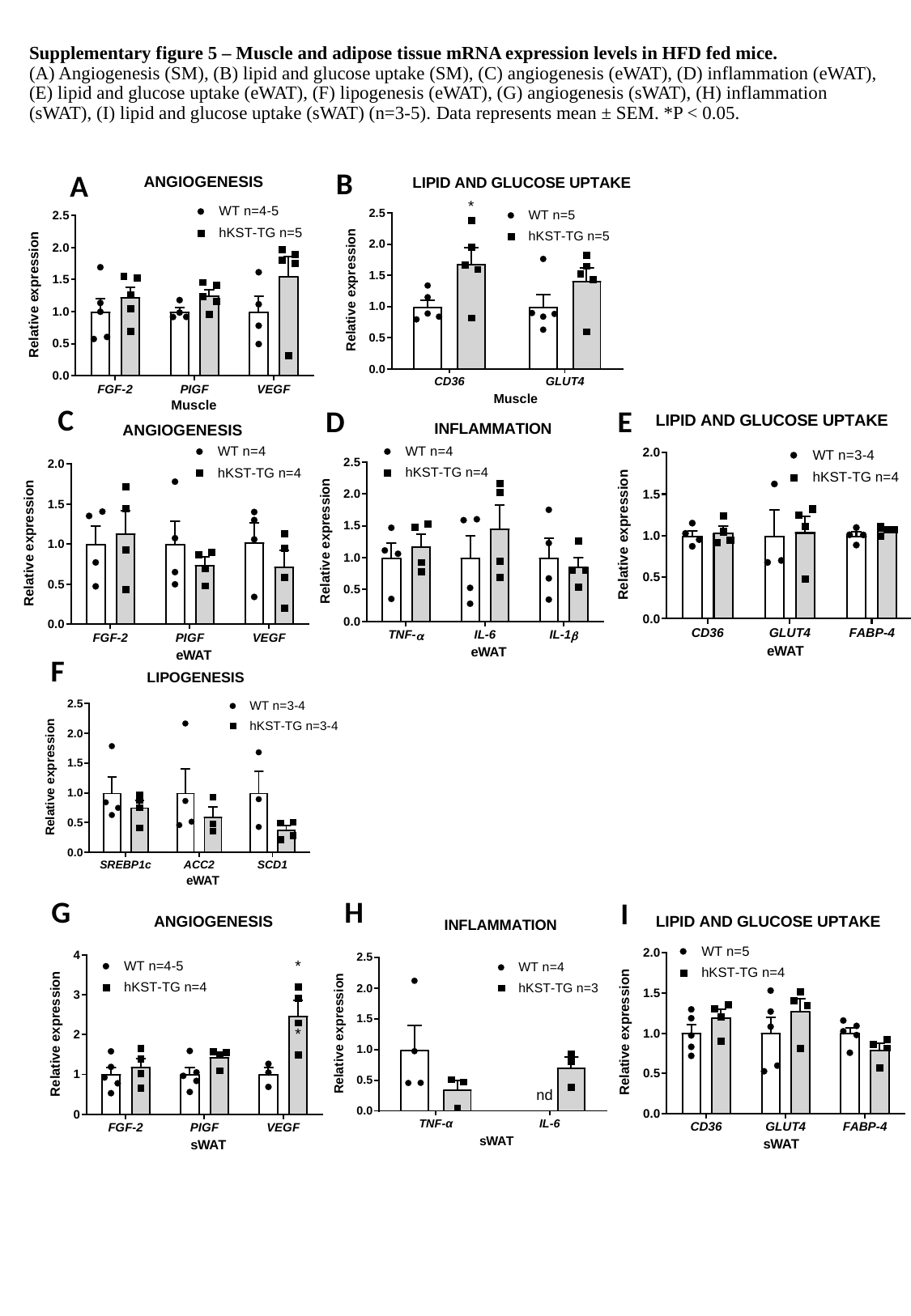

Supplementary figure 5 – Muscle and adipose tissue mRNA expression levels in HFD fed mice.
(A) Angiogenesis (SM), (B) lipid and glucose uptake (SM), (C) angiogenesis (eWAT), (D) inflammation (eWAT), (E) lipid and glucose uptake (eWAT), (F) lipogenesis (eWAT), (G) angiogenesis (sWAT), (H) inflammation (sWAT), (I) lipid and glucose uptake (sWAT) (n=3-5). Data represents mean ± SEM. *P < 0.05.
#
B
A
C
D
E
F
G
H
I

## Slide 6
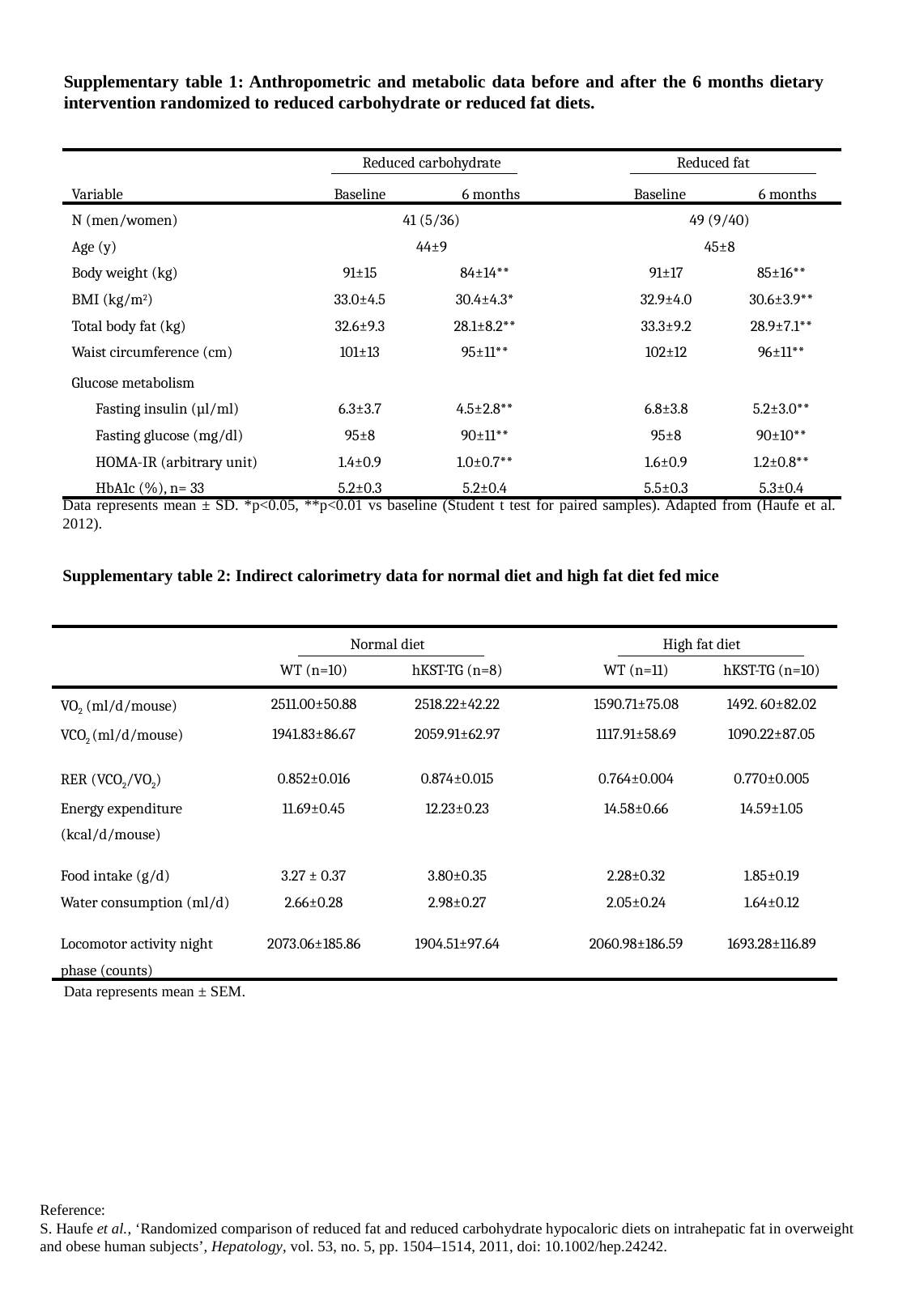

Supplementary table 1: Anthropometric and metabolic data before and after the 6 months dietary intervention randomized to reduced carbohydrate or reduced fat diets.
| | Reduced carbohydrate | | | | Reduced fat | | | | |
| --- | --- | --- | --- | --- | --- | --- | --- | --- | --- |
| Variable | Baseline | 6 months | | | Baseline | | | | 6 months |
| N (men/women) | 41 (5/36) | | | | | 49 (9/40) | | | |
| Age (y) | 44±9 | | | | | 45±8 | | | |
| Body weight (kg) | 91±15 | 84±14\*\* | | | | | 91±17 | 85±16\*\* | |
| BMI (kg/m2) | 33.0±4.5 | 30.4±4.3\* | | | | | 32.9±4.0 | 30.6±3.9\*\* | |
| Total body fat (kg) | 32.6±9.3 | 28.1±8.2\*\* | | | | | 33.3±9.2 | 28.9±7.1\*\* | |
| Waist circumference (cm) | 101±13 | 95±11\*\* | | | | | 102±12 | 96±11\*\* | |
| Glucose metabolism | | | | | | | | | |
| Fasting insulin (µl/ml) | 6.3±3.7 | 4.5±2.8\*\* | | | | | 6.8±3.8 | 5.2±3.0\*\* | |
| Fasting glucose (mg/dl) | 95±8 | 90±11\*\* | | | | | 95±8 | 90±10\*\* | |
| HOMA-IR (arbitrary unit) | 1.4±0.9 | 1.0±0.7\*\* | | | | | 1.6±0.9 | 1.2±0.8\*\* | |
| HbA1c (%), n= 33 | 5.2±0.3 | 5.2±0.4 | | | | | 5.5±0.3 | 5.3±0.4 | |
Data represents mean ± SD. *p<0.05, **p<0.01 vs baseline (Student t test for paired samples). Adapted from (Haufe et al. 2012).
Supplementary table 2: Indirect calorimetry data for normal diet and high fat diet fed mice
| | Normal diet | | | High fat diet | |
| --- | --- | --- | --- | --- | --- |
| | WT (n=10) | hKST-TG (n=8) | | WT (n=11) | hKST-TG (n=10) |
| VO2 (ml/d/mouse) | 2511.00±50.88 | 2518.22±42.22 | | 1590.71±75.08 | 1492. 60±82.02 |
| VCO2 (ml/d/mouse) | 1941.83±86.67 | 2059.91±62.97 | | 1117.91±58.69 | 1090.22±87.05 |
| | | | | | |
| RER (VCO2/VO2) | 0.852±0.016 | 0.874±0.015 | | 0.764±0.004 | 0.770±0.005 |
| Energy expenditure (kcal/d/mouse) | 11.69±0.45 | 12.23±0.23 | | 14.58±0.66 | 14.59±1.05 |
| | | | | | |
| Food intake (g/d) | 3.27 ± 0.37 | 3.80±0.35 | | 2.28±0.32 | 1.85±0.19 |
| Water consumption (ml/d) | 2.66±0.28 | 2.98±0.27 | | 2.05±0.24 | 1.64±0.12 |
| | | | | | |
| Locomotor activity night phase (counts) | 2073.06±185.86 | 1904.51±97.64 | | 2060.98±186.59 | 1693.28±116.89 |
Data represents mean ± SEM.
Reference:
S. Haufe et al., ‘Randomized comparison of reduced fat and reduced carbohydrate hypocaloric diets on intrahepatic fat in overweight and obese human subjects’, Hepatology, vol. 53, no. 5, pp. 1504–1514, 2011, doi: 10.1002/hep.24242.

## Slide 7
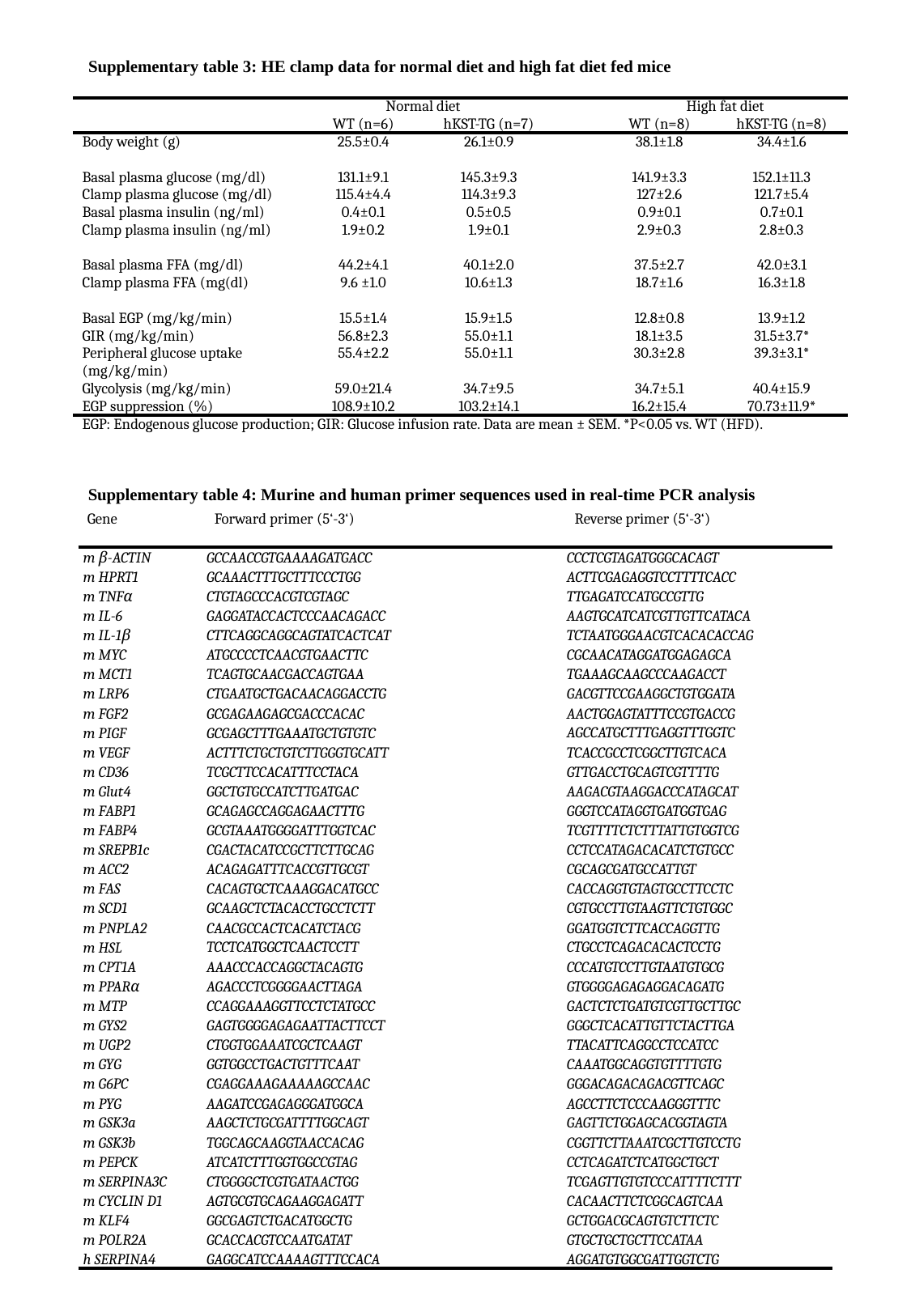

Supplementary table 3: HE clamp data for normal diet and high fat diet fed mice
| | Normal diet | | | High fat diet | |
| --- | --- | --- | --- | --- | --- |
| | WT (n=6) | hKST-TG (n=7) | | WT (n=8) | hKST-TG (n=8) |
| Body weight (g) | 25.5±0.4 | 26.1±0.9 | | 38.1±1.8 | 34.4±1.6 |
| | | | | | |
| Basal plasma glucose (mg/dl) | 131.1±9.1 | 145.3±9.3 | | 141.9±3.3 | 152.1±11.3 |
| Clamp plasma glucose (mg/dl) | 115.4±4.4 | 114.3±9.3 | | 127±2.6 | 121.7±5.4 |
| Basal plasma insulin (ng/ml) | 0.4±0.1 | 0.5±0.5 | | 0.9±0.1 | 0.7±0.1 |
| Clamp plasma insulin (ng/ml) | 1.9±0.2 | 1.9±0.1 | | 2.9±0.3 | 2.8±0.3 |
| | | | | | |
| Basal plasma FFA (mg/dl) | 44.2±4.1 | 40.1±2.0 | | 37.5±2.7 | 42.0±3.1 |
| Clamp plasma FFA (mg(dl) | 9.6 ±1.0 | 10.6±1.3 | | 18.7±1.6 | 16.3±1.8 |
| | | | | | |
| Basal EGP (mg/kg/min) | 15.5±1.4 | 15.9±1.5 | | 12.8±0.8 | 13.9±1.2 |
| GIR (mg/kg/min) | 56.8±2.3 | 55.0±1.1 | | 18.1±3.5 | 31.5±3.7\* |
| Peripheral glucose uptake (mg/kg/min) | 55.4±2.2 | 55.0±1.1 | | 30.3±2.8 | 39.3±3.1\* |
| Glycolysis (mg/kg/min) | 59.0±21.4 | 34.7±9.5 | | 34.7±5.1 | 40.4±15.9 |
| EGP suppression (%) | 108.9±10.2 | 103.2±14.1 | | 16.2±15.4 | 70.73±11.9\* |
| EGP: Endogenous glucose production; GIR: Glucose infusion rate. Data are mean ± SEM. \*P<0.05 vs. WT (HFD). | | | | | |
Supplementary table 4: Murine and human primer sequences used in real-time PCR analysis
| Gene | Forward primer (5‘-3‘) | Reverse primer (5‘-3‘) |
| --- | --- | --- |
| m β-ACTIN | GCCAACCGTGAAAAGATGACC | CCCTCGTAGATGGGCACAGT |
| m HPRT1 | GCAAACTTTGCTTTCCCTGG | ACTTCGAGAGGTCCTTTTCACC |
| m TNFα | CTGTAGCCCACGTCGTAGC | TTGAGATCCATGCCGTTG |
| m IL-6 | GAGGATACCACTCCCAACAGACC | AAGTGCATCATCGTTGTTCATACA |
| m IL-1β | CTTCAGGCAGGCAGTATCACTCAT | TCTAATGGGAACGTCACACACCAG |
| m MYC | ATGCCCCTCAACGTGAACTTC | CGCAACATAGGATGGAGAGCA |
| m MCT1 | TCAGTGCAACGACCAGTGAA | TGAAAGCAAGCCCAAGACCT |
| m LRP6 | CTGAATGCTGACAACAGGACCTG | GACGTTCCGAAGGCTGTGGATA |
| m FGF2 | GCGAGAAGAGCGACCCACAC | AACTGGAGTATTTCCGTGACCG |
| m PIGF | GCGAGCTTTGAAATGCTGTGTC | AGCCATGCTTTGAGGTTTGGTC |
| m VEGF | ACTTTCTGCTGTCTTGGGTGCATT | TCACCGCCTCGGCTTGTCACA |
| m CD36 | TCGCTTCCACATTTCCTACA | GTTGACCTGCAGTCGTTTTG |
| m Glut4 | GGCTGTGCCATCTTGATGAC | AAGACGTAAGGACCCATAGCAT |
| m FABP1 | GCAGAGCCAGGAGAACTTTG | GGGTCCATAGGTGATGGTGAG |
| m FABP4 | GCGTAAATGGGGATTTGGTCAC | TCGTTTTCTCTTTATTGTGGTCG |
| m SREPB1c | CGACTACATCCGCTTCTTGCAG | CCTCCATAGACACATCTGTGCC |
| m ACC2 | ACAGAGATTTCACCGTTGCGT | CGCAGCGATGCCATTGT |
| m FAS | CACAGTGCTCAAAGGACATGCC | CACCAGGTGTAGTGCCTTCCTC |
| m SCD1 | GCAAGCTCTACACCTGCCTCTT | CGTGCCTTGTAAGTTCTGTGGC |
| m PNPLA2 | CAACGCCACTCACATCTACG | GGATGGTCTTCACCAGGTTG |
| m HSL | TCCTCATGGCTCAACTCCTT | CTGCCTCAGACACACTCCTG |
| m CPT1A | AAACCCACCAGGCTACAGTG | CCCATGTCCTTGTAATGTGCG |
| m PPARα | AGACCCTCGGGGAACTTAGA | GTGGGGAGAGAGGACAGATG |
| m MTP | CCAGGAAAGGTTCCTCTATGCC | GACTCTCTGATGTCGTTGCTTGC |
| m GYS2 | GAGTGGGGAGAGAATTACTTCCT | GGGCTCACATTGTTCTACTTGA |
| m UGP2 | CTGGTGGAAATCGCTCAAGT | TTACATTCAGGCCTCCATCC |
| m GYG | GGTGGCCTGACTGTTTCAAT | CAAATGGCAGGTGTTTTGTG |
| m G6PC | CGAGGAAAGAAAAAGCCAAC | GGGACAGACAGACGTTCAGC |
| m PYG | AAGATCCGAGAGGGATGGCA | AGCCTTCTCCCAAGGGTTTC |
| m GSK3a | AAGCTCTGCGATTTTGGCAGT | GAGTTCTGGAGCACGGTAGTA |
| m GSK3b | TGGCAGCAAGGTAACCACAG | CGGTTCTTAAATCGCTTGTCCTG |
| m PEPCK | ATCATCTTTGGTGGCCGTAG | CCTCAGATCTCATGGCTGCT |
| m SERPINA3C | CTGGGGCTCGTGATAACTGG | TCGAGTTGTGTCCCATTTTCTTT |
| m CYCLIN D1 | AGTGCGTGCAGAAGGAGATT | CACAACTTCTCGGCAGTCAA |
| m KLF4 | GGCGAGTCTGACATGGCTG | GCTGGACGCAGTGTCTTCTC |
| m POLR2A | GCACCACGTCCAATGATAT | GTGCTGCTGCTTCCATAA |
| h SERPINA4 | GAGGCATCCAAAAGTTTCCACA | AGGATGTGGCGATTGGTCTG |
